# Phosphatidylglucoside regulates apoptosis of human neutrophilic lineage cells

**DOI:** 10.3389/fimmu.2025.1597423

**Published:** 2025-05-27

**Authors:** Noriko Yokoyama, Roudy Chiminch Ekyalongo, Madoka Kage, Kei Hanafusa, Hitoshi Nakayama, Yoshio Hirabayashi, Kenji Takamori, Kazuhisa Iwabuchi

**Affiliations:** ^1^ Institute for Environmental and Gender Specific Medicine, Juntendo University Graduate School of Medicine, Chiba, Japan; ^2^ Laboratory of Dermatological Physiology, Faculty of Pharmacy and Pharmaceutical Sciences, Josai University, Saitama, Japan; ^3^ Laboratory of Biochemistry, Faculty of Pharmacy, Juntendo University, Urayasu, Chiba, Japan; ^4^ Infection Control Nursing, Juntendo University Graduate School of Health Care and Nursing, Chiba, Japan; ^5^ Laboratory of Biochemistry, Juntendo University Faculty of Health Care and Nursing, Chiba, Japan; ^6^ Cellular Informatics Lab, RIKEN, Saitama, Japan

**Keywords:** phosphatidylglucoside, acute myeloid leukemia cells, apoptosis, cell death, differentiation, ATRA, KG1 cells

## Abstract

Apoptosis plays a fundamental role in the regulation of immune responses mediated by neutrophils. Phosphatidylglucoside (PtdGlc), a glycosylated phospholipid abundantly expressed on the surface of human neutrophils, has been implicated in promoting both cellular differentiation and apoptosis. In the acute myeloid leukemia (AML) cell line HL-60, PtdGlc expression increases during differentiation, and treatment with the anti-PtdGlc monoclonal antibody DIM21 induces early apoptosis. To further investigate the role of PtdGlc in neutrophilic lineage cells, we examined three AML cell lines: HL-60 (AML-M2/M3), KG1 (AML-M1), and KG1a (AML-M0). PtdGlc was highly expressed in HL-60 and KG1 cells but was absent in KG1a cells. Both HL-60 and KG1 cells exhibited early apoptosis following DIM21 treatment, whereas KG1a cells remained resistant regardless of differentiation status. Notably, in KG1 cells, DIM21 induced late-stage apoptosis specifically after ATRA-mediated differentiation, and co-treatment with ATRA and DIM21 significantly enhanced this apoptotic response. Mechanistic analysis revealed that this process was independent of NADPH oxidase and Fas signaling, as neither a reactive oxygen species inhibitor nor a neutralizing anti-Fas antibody altered the apoptotic outcome. Instead, DIM21 activated caspase-3 and caspase-8, suggesting that PtdGlc mediates apoptosis through a caspase-dependent, but NADPH oxidase- and Fas-independent, pathway. Collectively, these findings provide new insight into the apoptotic signaling function of PtdGlc in neutrophilic lineage cells and highlight its potential as a novel therapeutic target in AML.

## 1 Introduction

Neutrophils typically undergo spontaneous apoptosis within 12–24 hours of entering the bloodstream. Apoptotic neutrophils are phagocytosed by macrophages in a process known as efferocytosis, which helps maintain homeostasis by preventing the release of damaging acidic granules and proteases (1, 2). Macrophages involved in neutrophil clearance secrete granulocyte colony-stimulating factor (G-CSF), which promotes neutrophil differentiation and maturation, thereby maintaining neutrophil homeostasis (3). However, when neutrophils are not properly cleared, they may undergo necrosis or necroptosis, leading to tissue damage and chronic inflammation (4). Despite the critical role of neutrophil apoptosis in immune regulation, the mechanisms underlying this process remain unclear.

Phosphatidyl-glucoside (PtdGlc), a glycerophospholipid composed of a glucose molecule bound to phosphatidic acid, is expressed on the surface of human neutrophilic lineage cells (5, 6). PtdGlc was discovered as an unknown glucosylated phospholipid in human cord blood cells using a human anti-i monoclonal antibody GL-2 (5). GL2, a natural autoantibody with broad cross-reactivity, detected a similar but non-i-active glycolipid in the acute myeloid leukemia (AML) cell line HL-60. A recombinant Fab fragment of GL-2, rGL-7, was generated via *in vitro* transformation with Epstein-Barr virus. rGL-7 showed all-trans retinoic acid (ATRA)-like neutrophilic differentiation activity in HL-60 cells (7). However, rGL-7 bound to not only PtdGlc but also several other molecules. To overcome the lack of specificity, monoclonal antibody DIM21 was developed against PtdGlc using the detergent-insoluble membrane fraction of HL-60 cells (8). Like rGL-7 and GL-2, DIM21 induces neutrophil apoptosis through the Fas/caspase signaling pathway (7). PtdGlc expression increases with neutrophil maturation (6). It plays a role in the differentiation of human AML cell line HL-60 (9) and has been identified as a marker of neural stem cells in adult mice (10). Accordingly, PtdGlc expression and synthesis have attracted interest regarding potential involvement in the differentiation and maturation of myeloid cells. In addition, PtdGlc is deacylated by secretory phospholipase A_2_, releasing a lyso form (lysoPtdGlc) into the extracellular space. LysoPtdGlc functions as a spatial axon guidance molecule by interacting with G protein-coupled receptor (GPR) 55 (11). Recently, lysoPtdGlc has been shown to act as a GPR55-mediated chemotactic molecule for human monocytes and macrophages (12). These observations suggest the PtdGlc/lysoPtdGlc/GPR55 axis may be associated with neutrophil homeostasis.

AML is a malignancy characterized by poor differentiation and resistance to apoptosis, both of which contribute to its aggressive nature. As the most common form of acute leukemia in adults, AML displays uncontrolled cell growth and abnormal differentiation regulated by complex signaling pathways. To facilitate research, several AML cell lines have been established, among which HL-60 (AML-M2/M3) derived from a female patient, is used to study human myeloid differentiation (13). When treated with dimethyl sulfoxide (DMSO), HL-60 cells, originally neutrophilic promyelocytes, differentiate into neutrophil-like cells (14), providing insights into myeloid regulation. Although this DMSO-induced differentiation involves wild-type Kras and Wnt/β-catenin signaling pathways (15), the underlying mechanisms remain unclear.

In contrast, KG1 cell line (AML-M0/M1) represents an early progenitor stage with more primitive features than HL-60 cells (16). KG1 cells are less prone to differentiation and retain characteristics of undifferentiated hematopoietic progenitors and myeloblasts, making them a useful model for studying stem cell-like properties in AML. KG1a cells, a subclone of KG1, show high resistance to differentiation and apoptosis induction (17, 18). The anti-PtdGlc monoclonal antibody DIM21 has been shown to induce neutrophil apoptosis via Fas-mediated death signaling (19). To investigate apoptosis in neutrophilic lineage cells and the role of PtdGlc in this process, we characterized PtdGlc-mediated responses in HL-60, KG1, and KG1a cells. Our findings suggest PtdGlc expression on myeloid cells is important for their apoptosis.

## 2 Materials and methods

### 2.1 Materials

DMSO, ATRA, RPMI 1640, ATO, and trypan blue solution were from Sigma-Aldrich (St. Louis, MO, USA). The complete protease inhibitor cocktail (cOmplete) was from Roche Diagnostics (Tokyo, Japan). Propidium iodide (PI) was from Dojindo (Kumamoto, Japan). Immobilon-P PVDF membrane was from Millipore (Bedford, MA, USA). Caspase-8 inhibitor (Z-IETD-FMK) and caspase-9 inhibitor (Z-LEHD-FMK) were from MBL (Tokyo, Japan), and caspase-3 inhibitor (DEVD-CHO) was from Merck (Rahway, NJ, USA). Akt-1/2 inhibitor was from Abcam (San Francisco, CA, USA). Diphenyleneiodonium (DPI) was from Cayman Chemical (Ann Arbor, MI, USA). Alexa Fluor 488 monoclonal antibody labeling kit was from Invitrogen (Thermo Fisher Scientific, Waltham, MA, USA). The Situ Cell Death Detection Kit was from Roche (Roche Molecular Biochemistry, Indianapolis, IN, USA). BD Pharmingen FITC BrdU Flow kit was from Becton Dickinson (San Jose, CA, USA), and Wright and Giemsa solutions were from Muto Pure Chemical (Tokyo, Japan).

### 2.2 Antibodies

Mouse anti-PtdGlc monoclonal IgM DIM21 was prepared as described (8, 20). Additional antibodies used in this study are listed in **Supplementary Table S1**.

### 2.3 Cell culture

HL-60 cells were purchased from American Type Culture Collection (ATCC; Manassas, VA). KG1 and KG1a cells were kindly provided by Dr. Yoshio Hirabayashi (RIKEN, Saitama, Japan). Human leukemia cell lines (HL-60, KG1a, and KG1) were cultured in RPMI 1640 medium supplemented with 1% penicillin/streptomycin at the following concentrations: 10% fetal bovine serum (FBS) for HL-60 and 20% FBS for KG1 and KG1a. HL-60 cells were differentiated into neutrophilic lineage cells (DHL-60) by culture in RPMI medium containing 1.3% DMSO for 4 days. In some experiments, HL-60, KG1, and KG1a cells were treated with either 1.3% DMSO or 1 μM ATRA in RPMI medium for five or six days.

### 2.4 Annexin V-binding assay

Various leukemia cells (1 x 10^6^ cells/ml) were incubated in DMEM/F12 medium containing 10% FBS with either 4–5 μg/ml IgM or DIM21 antibody for 4 h at 37°C. In some experiments, cells were pretreated with various distinct reagents for four or six days, followed by incubation with either IgM, DIM21 antibody, or anti-Fas antibody (CH-11, 5 μg/ml) for 4 h. To assess the effects of caspase inhibition, cells were pretreated with 10 μM caspase-3 inhibitor, 10 μM caspase-8 inhibitor, or 10 μM caspase-9 inhibitor for 16 h before apoptosis was induced by adding either IgM or DIM21 antibody for 4 h. Cells were harvested and washed twice with Annexin V-binding buffer (10 mM HEPES 2-[4-(2-hydroxyethyl)-1-piperazinyl] ethane sulfonic acid (Hepes), 140 mM NaCl, 2.5 mM CaCl_2_, pH 7.4). Cells were incubated with Alexa 488- conjugated Annexin V for 15 min at room temperature, washed twice with Annexin V-binding buffer, and analyzed by flow cytometry with or without 1 μg/ml of PI. To assess the involvement of ROS, KG1 cells were pretreated with 10 μM DPI or vehicle (DMSO, 2.83 μl) for 1 h, followed by Annexin V-binding assay. To evaluate the role of AKT, KG1 cells were treated with 5 μM AKT1/2 inhibitor or 1 μl vehicle in the presence of either IgM or DIM21 for 4 h, followed by Annexin V-binding assay. To investigate the effect of Fas antagonist antibody (ZB4) on anti-Fas antibody-induced apoptosis, cells were incubated with ZB4 (10 μg/ml) for 2 h before analysis.

### 2.5 Analysis of surface expression of Fas, CD11b, CD14, CD38, and PtdGlc by flow cytometry

Cells were untreated or treated with the indicated reagents. After collection and washing with PBS, cells (2.5 x 10^6^ cells/ml) were stained with either PE-labeled anti-Fas, PE-labeled anti-CD11b, PE-labeled anti-CD14, Alexa 488-conjugated anti-CD38, or Alexa 488-conjugated anti-PtdGlc antibodies (DIM21) for 30 min on ice. Following incubation, cells were washed twice with PBS and suspended in PBS. Expressions of Fas, CD11b, CD14, CD38, and PtdGlc were analyzed by flow cytometry (Flow Cytometry Calibur, BD Biosciences). For negative controls, cells were stained with either PE-labeled anti-IgG κ, Alexa 488-conjugated IgG, or Alexa 488-conjugated IgM. Data were analyzed using CellQuest Pro software (BD Biosciences).

### 2.6 Granulocytic profiling

Cells (1 x 10^5^) were seeded onto slides using a cytospin (500 rpm, 2 min), air-dried, and stained with Wright/Giemsa solutions. Samples were visualized using digital microscopy (Keyence, Bz-9000).

### 2.7 Analysis of cell-cycle progression and proliferation

Cells were treated with 5 μg/ml IgM, 5 μg/ml DIM21, ATRA (1 μM), ATRA plus IgM, or ATRA plus DIM21 for four days, followed by 1 h BrdU pulse at 37°C in a humidified atmosphere with 5% CO2. Cells were prepared following the manufacturer’s instructions and analyzed by flow cytometry. DIM21 induced not only neutrophilic differentiation but also apoptosis in leukemia cells. Cell cycle distribution (G0/G1, S, and G2/M phases) was assessed based on DNA content after staining with 7-amino-actinomycin D (7-AAD).

### 2.8 TUNEL assay

HL-60 cells (1 x 10^5^/ml) were treated without or with 0.5 μg/ml mouse IgM or DIM21 in the presence of either 1 μM ATRA or ethanol as a solvent control (at a final concentration of 0.02v/v%) for seven days. DNA strand breaks were detected using the *In Situ* Cell Death Detection kit (Roche Molecular Biochemistry, Indianapolis, IN, USA) following the manufacturer’s instructions. Briefly, cells (2 x 10^6^ cells/ml) were washed with PBS, fixed with 2% paraformaldehyde at 25°C for 1 h, and permeabilized with 0.1% Triton-X-100 in 0.1% sodium citrate. After washing with PBS, fragmented DNA was detected using the labeling solution with or without terminal transferase. Images were captured using TCS STED CW super-resolution confocal microscope with 63× objective lens (Leica).

### 2.9 Cell lysates

HL-60, KG1a, and KG1 cells were either untreated or treated with 1 μM ATRA, 2 μg/ml DIM21, or their combination for six days. Cells were washed with PBS and lysed in RIPA buffer (150 mM NaCl, 5 mM EDTA, 1% NP-40, 0.25% sodium deoxycholate, 2 mM sodium orthovanadate, 0.05% SDS, 50 mM Tris-HCl, pH 7.2) containing protease inhibitors. Lysates were obtained by centrifugation at 20,800 x g for 10 min, and the supernatant was collected. Proteins (20 μg) were subjected to SDS-PAGE and analyzed by Western blotting.

### 2.10 Immunoblotting

Cell lysates were subjected to SDS-PAGE, and proteins were transferred to an Immobilon membrane and incubated with the indicated antibodies. Bands were detected with horseradish peroxidase-conjugated secondary antibody. Immunocomplexes were detected by ECL chemiluminescence (Pierce Biotechnology, Rockland, IL, USA). Bands detected by ECL chemiluminescence were scanned, and intensities were quantified using the ImageJ program 1.50b (US National Institutes of Health; https://rsb.info.nih.gov/ij/).

### 2.11 Statistical analysis

Data are presented as mean ± SEM. Normality and homogeneity of variance were assessed prior to statistical analysis. Statistical significance was evaluated using one-way or two-way ANOVA, followed by Tukey’s or Sidak’s multiple comparisons test, as appropriate. All analyses were conducted using GraphPad Prism version 10 (GraphPad Software, San Diego, CA, USA). A p-value of <0.05 was considered statistically significant.

## 3 Results

### 3.1 Neutrophilic differentiation of HL-60 cells induced by DMSO or ATRA treatment leads to apoptosis via PtdGlc

#### 3.1.1 PtdGlc expression increased during DMSO- or ATRA-induced differentiation of HL-60 cells

**Figure 1:**
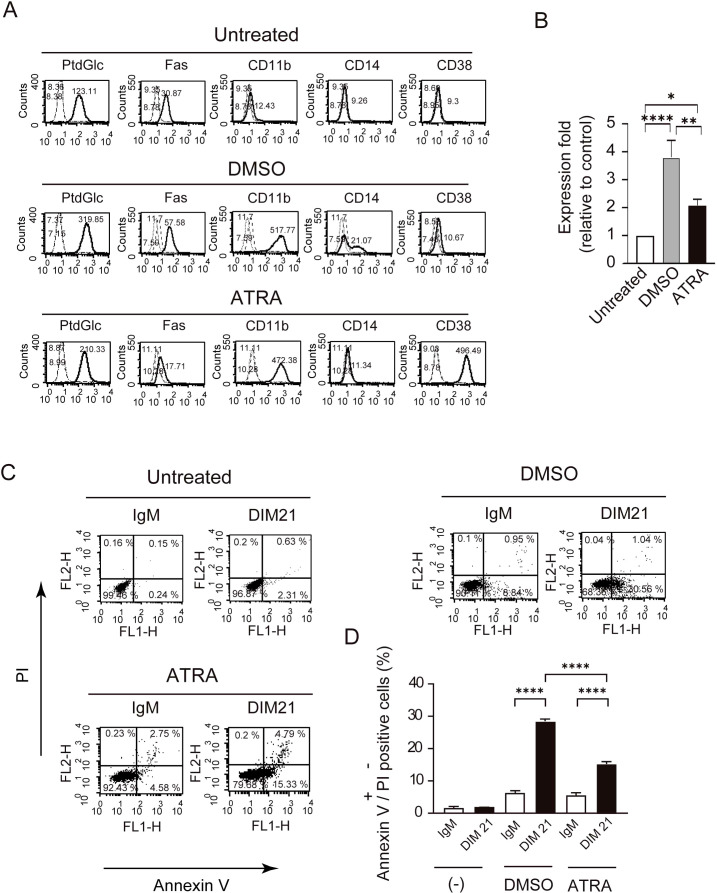
Differentiation of HL-60 cells by DMSO or ATRA and apoptosis induction by DIM21. Induction of PtdGlc and CD11b expression by DMSO or ATRA treatment. **(A)** HL-60 cells were treated with either 1.3% DMSO or 1 μM ATRA for six days. Treated and untreated cells (2.5 × 10^6^ cells/ml) were incubated with Alexa488-conjugated DIM21, Alexa488-conjugated mouse IgM, PE-anti-human Fas, PE-IgG1 κ, PE-anti-human CD11b, PE-anti-human CD14, Alexa488-conjugated mouse IgG1 κ, or Alexa488-conjugated anti-human CD38. After staining, cells were washed with PBS and analyzed by flow cytometry. Histograms show PtdGlc, Fas, CD11b, CD14, and CD38 staining (thick lines), with negative controls (Alexa488-IgM, PE-IgG1 κ, or Alexa488-mouse IgG) represented by broad dashed lines (—) and untreated cells by narrow dashed lines (^……^). Numbers indicate geometric mean fluorescence intensity. Representative results of 11 independent experiments are shown. **(B)** Induction of PtdGlc expression in DMSO- or ATRA-treated HL-60 cells. Ratios of PtdGlc expression relative to untreated cells are shown. Data represent the mean ± SE of 6 to 7 independent experiments. Statistical significance: *P < 0.05, **P < 0.005, ****P < 0.0001. **(C)** DIM21-induced apoptosis in differentiated HL-60 cells. HL-60 cells treated with DMSO or ATRA for six days were incubated with 4 μg/ml DIM21 or IgM (negative control) for 4 h. Cells were stained with Alexa488-conjugated Annexin V and analyzed by flow cytometry using propidium iodide (PI). Apoptotic cells (Annexin V^+^+PI^-^) appear in the lower right quadrant, and late apoptotic/dead cells (Annexin V^+^+PI^+^) in the upper right quadrant. Representative results from 5–17 independent experiments are shown. **(D)** Comparison of DIM21-induced apoptosis between DMSO- and ATRA-treated HL-60 cells. The percentages of Annexin V^+^+PI^-^ cells (white bars) and Annexin V^+^+PI^+^ cells (black bars) were quantified after treatment with IgM or DIM21. Data represent the mean ± SE of 5–17 independent experiments. ****P < 0.0001.

HL-60 cells are known to undergo neutrophilic differentiation upon treatment with DMSO or ATRA (14, 21, 22). Undifferentiated HL-60 cells did not express CD11b, a well-established differentiation marker, on their plasma membranes (**Figure 1A**). Consistent with previous reports , CD11b, CD14, and CD38 were detected on HL-60 cells following treatment with DMSO or ATRA (**Figure 1A**) (23). In addition to these markers, Fas (CD95) was expressed upon differentiation with ATRA or DMSO (24). Under these conditions, PtdGlc was already present on undifferentiated cells, and its expression levels increased 3.80 ± 0.6-fold (mean ± SE, n= 6) in DMSO treated-cells and 2.08 ± 0.21-fold in ATRO-treated cells (**Figure 1B**). PtdGlc expression in human blood neutrophils was significantly higher than in HL-60 cells (**Supplementary Figures S1A, B**).

#### 3.1.2 PtdGlc mediated apoptosis of differentiated HL-60 cells by DMSO or ATRA treatment

PtdGlc has been implicated in the Fas/caspase signaling pathway-dependent apoptosis of neutrophils (19). Apoptosis occurs in distinct stages, early and late, each characterized by specific biochemical and morphological changes. Early apoptotic cells express phosphatidylserine, whereas late apoptotic cells stain with both Annexin V and PI (25). Similar to human neutrophils (19), incubation with DIM21, a monoclonal anti-PtdGlc IgM, for 4 h induced early apoptosis in both DMSO- and ATRA-differentiated HL-60 cells (**Figures 1C, D**).

### 3.2 PtdGlc promoted differentiation and late apoptosis of HL-60 cells in ATRA differentiation

#### 3.2.1 DIM21 had minimal effect on HL-60 cell differentiation

**Figure 2:**
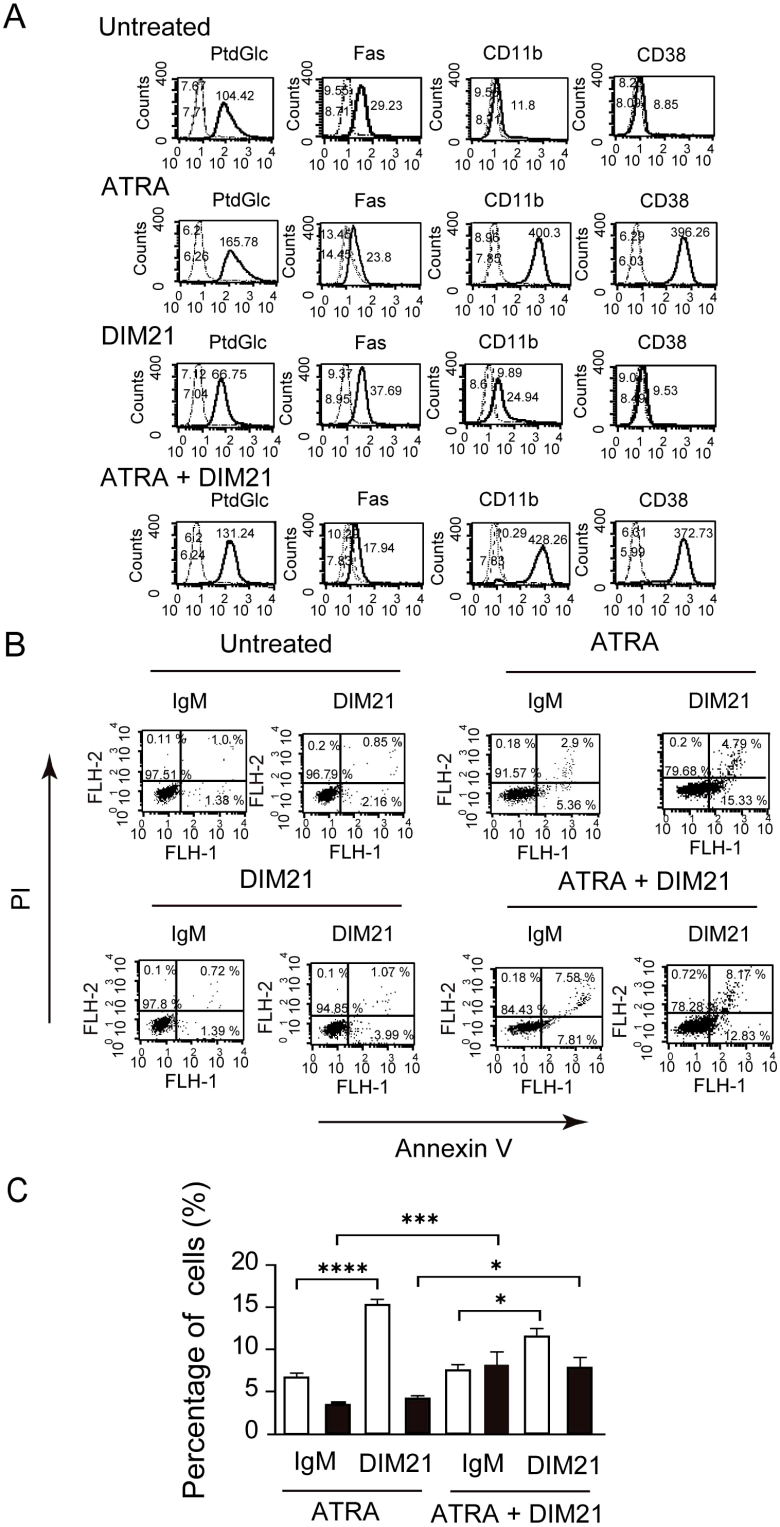
Multi-molecule expression and DIM21-induced apoptosis in HL-60 cells treated with ATRA or ATRA plus DIM21. **(A)** Upregulation of PtdGlc, CD11b, and CD38 expression in ATRA-treated HL-60 cells. HL-60 cells were treated with ATRA (1 μM), DIM21 (2 μg/ml), or ATRA plus DIM21 (ATRA + DIM21) for six days, and the expression of various molecules was analyzed. Treated and untreated cells (2.5 × 10^6^ cells/ml) were incubated with Alexa488-conjugated DIM21, Alexa488-conjugated mouse IgM, PE-anti-human Fas, PE-IgG1 κ, PE-anti-human CD11b, PE-anti-human CD14, Alexa488-conjugated mouse IgG, or Alexa488-conjugated anti-human CD38. After staining, cells were washed and analyzed by flow cytometry. Histograms show PtdGlc, Fas, CD11b, CD14, and CD38 expression (thick lines), with negative controls represented by broad dashed lines (—–) and untreated cells by narrow dashed lines (^……^). Numbers indicate geometric mean fluorescence intensity. Representative results from 9–12 independent experiments are shown. **(B)** DIM21-induced apoptosis in ATRA-treated HL-60 cells. HL-60 cells were treated with ATRA (1 μM), DIM21 (2 μg/ml), or ATRA + DIM21 for six days, followed by 4 μg/ml DIM21 or IgM (negative control) for 4 h. Cells were stained with Alexa488-conjugated Annexin V and analyzed by flow cytometry using PI. Apoptotic cells (Annexin V^+^+PI^-^) appear in the lower right quadrant, and late apoptotic/dead cells (Annexin V^+^+PI^+^) in the upper right quadrant. Representative results are from 9–17 independent experiments. **(C)** Comparison of DIM21-induced apoptosis in ATRA- and ATRA plus DIM21-treated HL-60 cells. HL-60 cells treated as described were analyzed for apoptosis. The percentages of early apoptotic cells (Annexin V^+^+PI^-^, white bars) and late apoptotic/dead cells (Annexin V^+^+PI^+^, black bars) were quantified after treatment with DIM21 or IgM. Data represent the mean ± SE of 9–17 independent experiments. Statistical significance: *P < 0.05, ***P < 0.005, ****P < 0.0001.

We previously demonstrated that recombinant Fab fragment of anti-glucosylated phospholipid antibody rGL-7 induced ATRA-like differentiation in HL-60 cells (7). However, HL-60 cells did not express CD38 after six days of treatment with monoclonal anti-PtdGlc IgM DIM21, though DIM21 slightly enhanced CD11b expression (**Figure 2A**). CD38 is upregulated during ATRA-induced HL-60 cell differentiation (26–28). To evaluate the effect of DIM21 on the expression of PtdGlc, Fas, CD11b, and CD38 in ATRA-induced HL-60 cells, we treated the cells with ATRA in the presence or absence of DIM21. DIM21 had little effect on the expression of these molecules in ATRA-treated HL-60 cells (**Figure 2A**). Vehicle (EtOH) and control IgM had no effect (**Supplementary Figure S2A**).

#### 3.2.2 DIM21 enhanced late apoptosis of ATRA-induced differentiated HL-60 cells

Apoptosis is a regulated process essential for tissue homeostasis, occurring in early and late stages, each with distinct cellular and molecular changes. DIM21 did not induce apoptosis in undifferentiated, IgM- or EtOH-treated HL-60 cells, despite the presence of PtdGlc (**Figure 2B**, **Supplementary Figure 2SB**). We compared the ratio of Annexin V^+^+PI^-^ (early apoptosis) with Annexin V^+^+ PI^+^ cells (late apoptosis/cell death) in ATRA-treated HL-60 cells with and without DIM21 (**Figure 2C**). ATRA-treated HL-60 cells in the presence of DIM21 showed increased late apoptosis compared to those cells treated with ATRA alone (**Figure 2C**). Thus, PtdGlc-mediated signaling may enhance apoptosis in ATRA-induced differentiated neutrophilic lineage cells.

#### 3.2.3 DIM21 enhanced cell cycle arrest during ATRA-induced differentiated HL-60 cells

**Figure 3:**
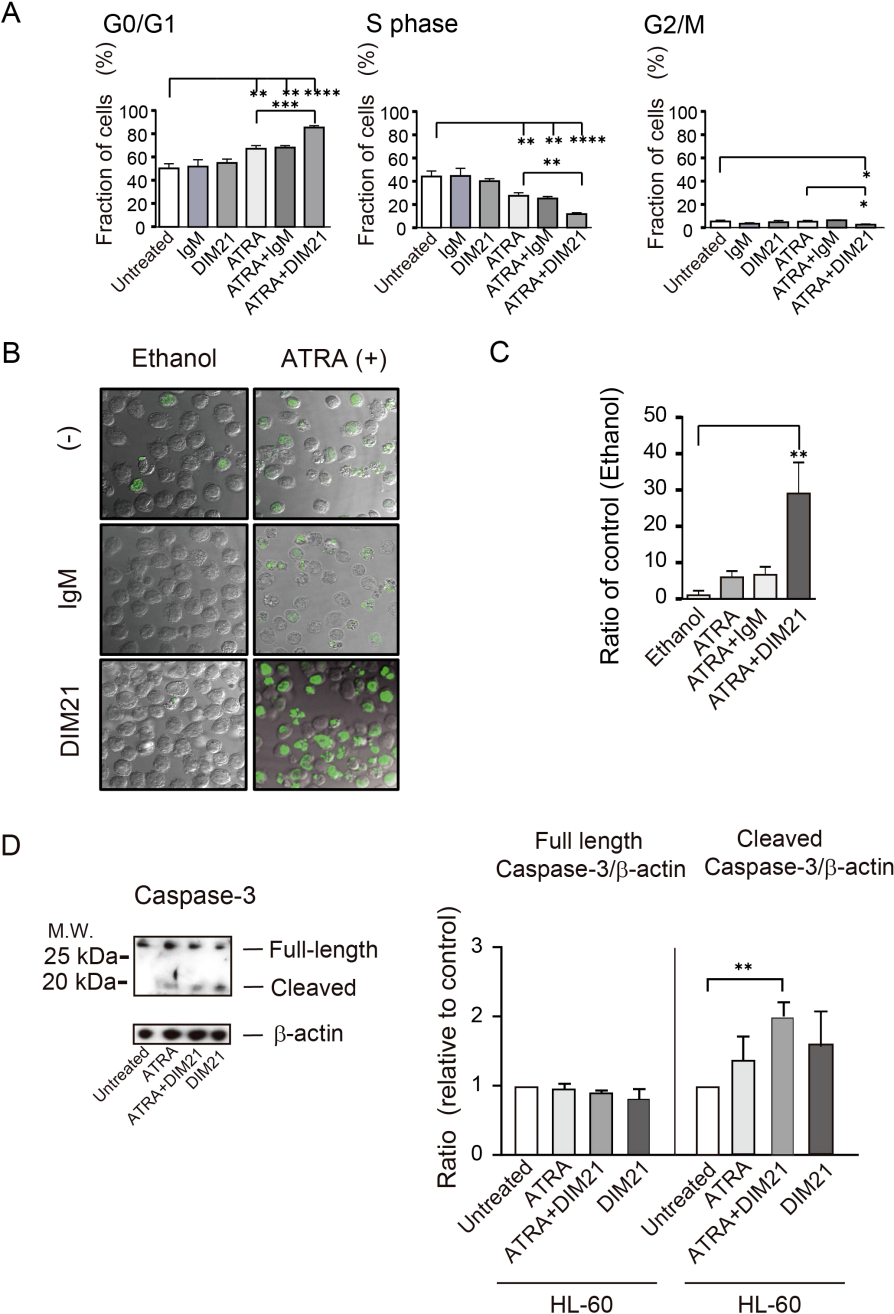
Characterization of DIM21-induced apoptosis in HL-60 cells treated with ATRA or ATRA plus DIM21. **(A)** ATRA-induced G0/G1 arrest in HL-60 cells. HL-60 cells were untreated or treated with 5 μg/ml IgM, 5 μg/ml DIM21, ATRA (1 μM), ATRA + IgM, or ATRA + DIM21 for four days. Cells were pulsed with BrdU for 1 h at 37°C in a 5% CO_2_ atmosphere and analyzed by flow cytometry. Cell cycle phases (G0/G1 and S) were identified based on DNA content using 7-AAD staining. Data represent the mean ± SE of 3 independent experiments. Statistical significance: *P < 0.05, **P < 0.005, ***P < 0.001, ****P < 0.0001. **(B)** Increased apoptosis in ATRA-treated HL-60 cells with DIM21 treatment. HL-60 cells (1 × 10^5^ cells/ml) were treated without or with 0.5μg/ml normal mouse IgM (IgM) or DIM21 in the presence of either 1 μM ATRA or ethanol as a solvent control (at a final concentration of 0.02v/v%) for seven days. After treatment, cells (2 × 10^7^ cells/ml) were subjected to TUNEL assay and analyzed using the confocal microscope (63× objective lens). Images show DNA fragmentation in ethanol as a vehicle-treated control (left column) and ATRA-treated cells (right column). Apoptotic cells (green fluorescence) and live cells were counted, and the apoptosis/live cell ratio was calculated. Representative images of 3 independent experiments are shown. **(C)** Quantification of apoptosis using a TUNEL assay by flow cytometry. Cells (10^4^ cells) were treated as described above and analyzed by flow cytometry to assess apoptosis. Data represent mean ± SE of 3 independent experiments. Statistical significance: **P < 0.005. **(D)** Caspase-3 activation in DIM21-induced apoptosis. HL-60 cells were treated with ATRA (1 μM), DIM21 (2 μg/ml), or ATRA + DIM21 for six days. Cells were harvested, washed, and lysed with RIPA buffer. Proteins (20 μg) were analyzed by SDS-PAGE and Western blotting using an anti-caspase-3 antibody. The expression ratio of caspase-3 relative to untreated cells was calculated. Representative images and quantitative data (mean ± SE) from 3 independent experiments are shown. Statistical significance: **P < 0.005.

The cell cycle of neutrophils is highly regulated during differentiation, with distinct changes at various phases (29). We analyzed the effect of DIM21 on the cell cycle of ATRA-treated HL-60 cells using DNA content analysis following 7-AAD staining. ATRA treatment increased the proportion of G0/G1-phase cells, decreasing S-phase cells compared to untreated cells (**Figure 3A**), suggesting ATRA induces G0/G1 arrest during HL-60 cell differentiation. DIM21 alone had no significant anti-proliferative effect on HL-60 cells. However, it enhanced ATRA-induced G0/G1 arrest and significantly reduced the proportion of G2/M-phase cells in ATRA-treated cells. ATRA inhibited cell growth by blocking cell cycle progression at the G1-phase, consistent with previous reports (30).

Enhancement of DIM21-induced apoptosis in ATRA-treated HL-60 cells was confirmed by TUNEL assay (**Figure 3B**). Membrane blebbing and nuclear and cytoplasmic shrinkage were greatly enhanced in cells treated with ATRA plus DIM21 but not in those treated with ATRA plus IgM or ATRA alone (**Figure 3B**). Flow cytometric TUNEL assay produced similar results (**Figure 3C**). TUNEL-positive apoptotic cells increased exponentially with ATRA plus DIM21 treatment (**Figure 3C**). Our results suggest that HL-60 cells differentiated by ATRA undergo apoptosis in the presence of DIM21 (**Figures 2B, C**, **3B, C**). These findings indicate PtdGlc is involved in HL-60 cell differentiation and apoptosis.

#### 3.2.4 Activation of the caspase cascades is linked to DIM21-induced apoptosis in ATRA-treated HL-60 cells

Our previous work demonstrated that Fas-dependent apoptosis in neutrophils involves large cluster formation and colocalization of Fas and PtdGlc on the plasma membrane (19). Caspase-3 is important in both intrinsic and extrinsic death pathways (31). It was reported that anti-Fas activation antibody induces caspase-3 and -8 but not -9 activation in DMSO- or ATRA-induced differentiated HL-60 cells (24). In the presence of DIM21, caspase-3 activation was already evident in ATRA-induced HL-60 cells (**Figure 3D**). Caspase-8 activation was also observed in ATRA-treated HL-60 cells in the presence of DIM21 (data not shown). These results indicate caspase-3 and -8 activation are associated with DIM21-induced apoptosis in ATRA-treated HL-60 cells.

### 3.3 Characterization of response to differentiation inducer in KG1a and KG1, expression of PtdGlc and induction of apoptosis

#### 3.3.1 Distinct surface expression of PtdGlc in KG1a and KG1 cells

**Figure 4:**
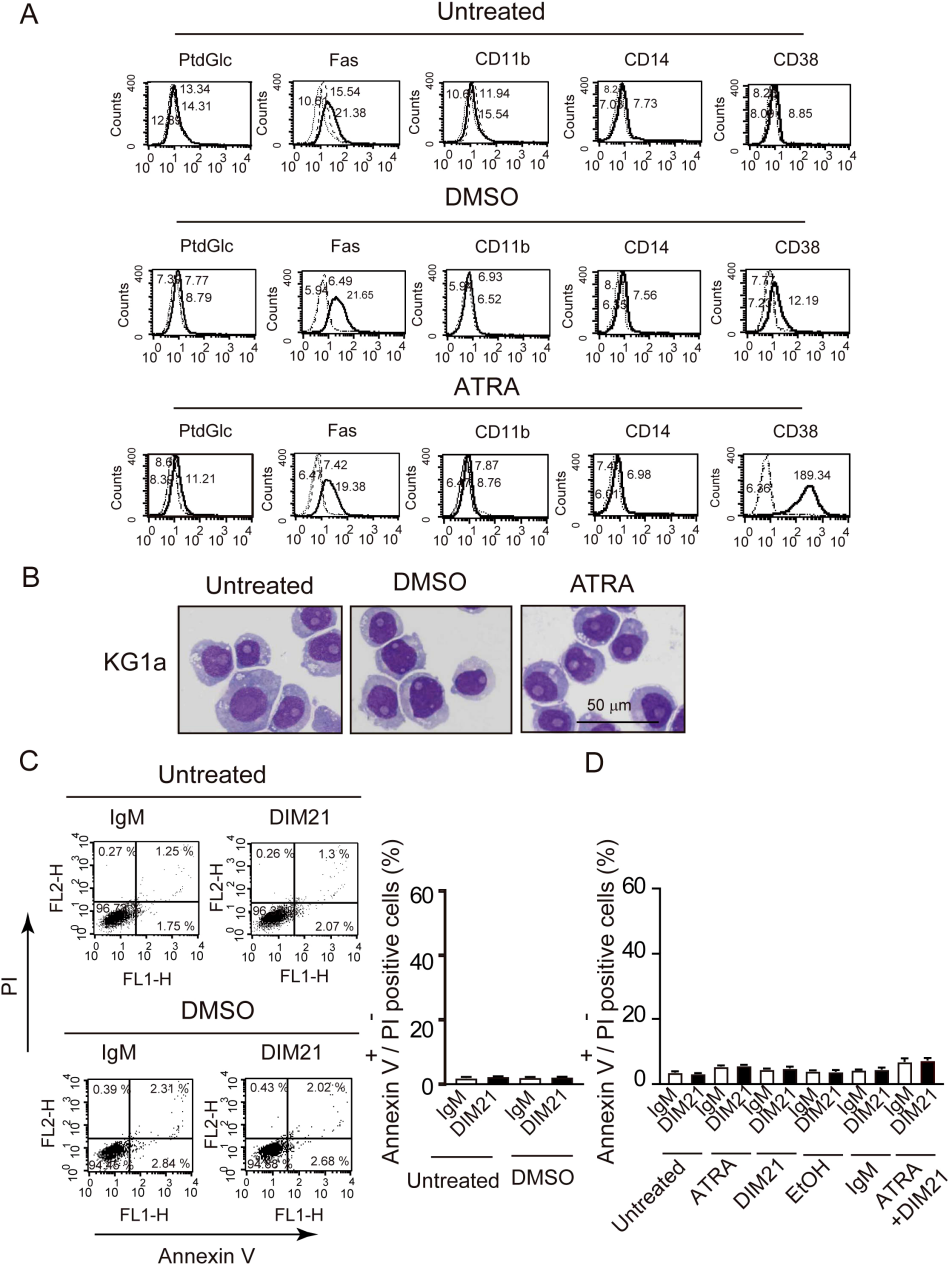
KG1a cells do not differentiate or undergo DIM21-induced apoptosis after DMSO or ATRA treatment. **(A)** Lack of multi-molecular expression changes in DMSO- or ATRA-treated KG1a cells. KG1a cells were untreated or treated with DMSO (1.3%) or ATRA (1 μM) for six days. Expression levels of PtdGlc, Fas, CD11b, CD14, and CD38 were measured by flow cytometry. Histograms show the following: thick lines represent PtdGlc, Fas, CD11b, CD14, and CD38; broad dashed lines (—–) represent negative controls (Alexa 488-mouse IgM or PE-IgG κ); and narrow dashed lines (^……^) represent untreated cells. Numbers represent geometric mean fluorescence intensity. Representative results from 5–14 independent experiments are shown. **(B)** Lack of morphological changes in DMSO- or ATRA-treated KG1a cells. KG1a cells were untreated or treated with DMSO (1.3%) or ATRA (1 μM) for six days. Cells were then prepared on glass slides using cytospin and stained with Wright-Giemsa solutions, followed by visualized using Keyence Bz-9000 microscope with 60× objective lens. Morphological evaluation revealed no significant changes between conditions. Representative results from 6 independent experiments are shown. **(C)** DIM21 fails to induce apoptosis in DMSO-treated KG1a cells. KG1a cells were treated with DMSO (1.3%) for six days, followed by incubation with either 4 μg/ml IgM (control) or DIM21 for 4 h. Early apoptotic cells (Annexin V^+^+PI^-^) appear in the lower right quadrant of the flow cytometry plots, and late apoptotic/dead cells (Annexin V^+^+PI^+^) appear in the upper right quadrant. Quadrant percentages reflect the distribution of cells in each phase. Data represent the mean ± SE of 5 independent experiments. **(D)** DIM21 fails to induce apoptosis in ATRA-treated KG1a cells. KG1a cells were treated with 1 μM ATRA, 2 μg/ml DIM2, ATRA + DIM21, vehicle (EtOH), or 2 μg/ml control IgM for six days. Cells (2.5 × 10^6^ cells/ml) were then incubated with 4 μg/ml DIM21 or IgM for 4 h. Apoptosis assays showed no significant induction of apoptosis. Early apoptotic cells (Annexin V^+^+PI^-^) appear in the lower right quadrant and late apoptotic/dead cells (Annexin V^+^+PI^+^) in the upper right quadrant. Representative results from 6–14 independent experiments are shown.

**Figure 5:**
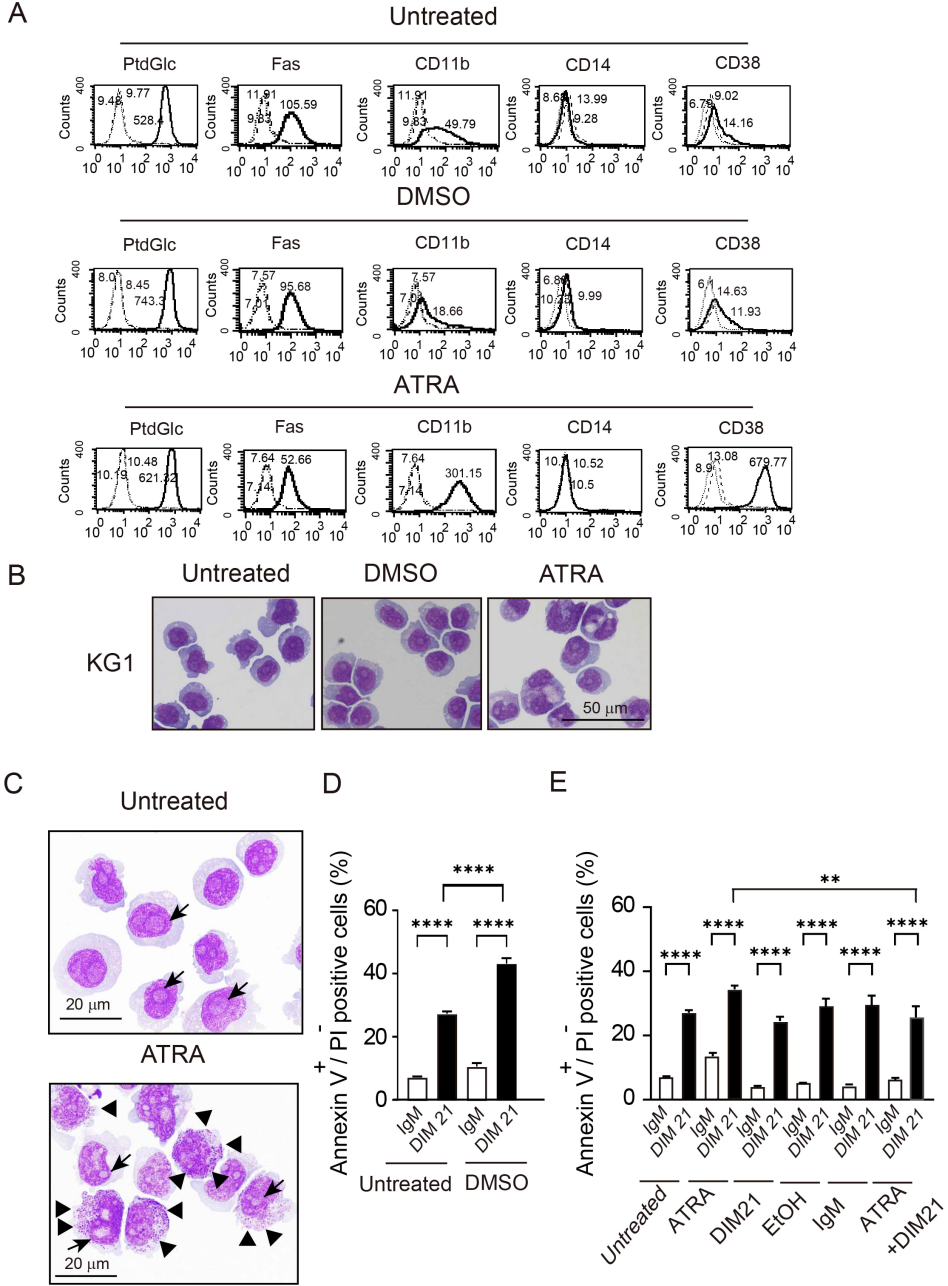
ATRA-induced differentiation and DIM21-induced apoptosis in KG1 cells. **(A)** Multi-molecule expression in DMSO- or ATRA-treated KG1 cells. KG1 cells were untreated or treated with DMSO (1.3%) or ATRA (1 μM) for six days. Expressions of PtdGlc, Fas, CD11b, CD14, and CD38 were assessed by flow cytometry. The thick lines represent the respective markers, broad dashed lines (—–) indicate negative controls (Alexa488-mouse IgM or PE-IgGκ) and narrow dashed lines (^……^) represent untreated cells. Numbers indicate geometric mean fluorescence intensity. Representative results of 14 independent experiments are shown. **(B)** Morphological changes in DMSO- or ATRA-treated KG1 cells. KG1 cells were treated as described above, washed with PBS, and mounted on slides by cytospin. Cells were stained with Wright-Giemsa solution, followed by visualized using Keyence Bz-9000 microscope with 60× objective lens. The results represent 6 independent experiments. **(C)** ATRA-induced differentiation with morphological characteristics. ATRA-treated KG1 cells showed azurophilic granules (arrowhead) and visible nucleoli (black arrow). Cells were visualized Keyence Bz-9000 microscope with 100× objective lens. Representative results of 6 independent experiments are shown. **(D)** DIM21-induced apoptosis in DMSO-treated KG1 cells. KG1 cells were treated with or without DMSO (1.3%) for six days and incubated with 4 μg/ml DIM21 or IgM (control) for 4 **(h)** Apoptotic cells (Annexin V^+^+PI^-^, lower right quadrant) and late apoptotic/dead cells (Annexin V^+^+PI^+^, upper right quadrant) were identified by flow cytometry. Data represent the mean ± SE of 20 independent experiments (****P < 0.0001). **(E)** DIM21-induced apoptosis in ATRA-treated KG1 cells. KG1 cells were treated without or with ATRA (1 μM), DIM21 (2 μg/ml), ATRA + DIM21, vehicle (EtOH) or control IgM (2 μg/ml) for six days, followed by incubation with 4 μg/ml DIM21 or IgM for 4 h. Apoptosis was evaluated as described in **(D)**. Data represent the mean ± SE of 14 independent experiments. **P < 0.01, ****P < 0.0001.

AML cells are characterized by impaired differentiation and resistance to apoptosis (32). Like HL-60 cells, KG1 cells can differentiate neutrophilic lineage cells under certain conditions (33, 34). In contrast, KG1a cells are resistant to differentiation inducers and chemotherapeutic agents, making them a useful model to study treatment-resistant AML (18, 35). To further analyze the role of PtdGlc in apoptosis within neutrophilic lineage cells, we investigated less mature KG1 and KG1a cells in comparison with HL-60 cells. Under our experimental conditions, ATRA failed to induce CD11b expression in ATRA-treated KG1a cells, although CD38 was expressed (**Figure 4A**, **Supplementary Figure S3A**). In contrast, as reported by Drach JG et al., (36, 37), ATRA-treated KG1 cells expressed both CD11b and CD38 (**Figure 5A**, **Supplementary Figure S3B**). However, KG1a cells did not express PtdGlc, regardless of treatment with differentiation inducers (**Figure 4A**), whereas KG1 cells expressed high levels of PtdGlc on their surfaces in untreated cells, and its expression was upregulated by DMSO or ATRA treatment (**Figure 5A**).

#### 3.3.2 ATRA induced morphological changes in KG1 but not KG1a cells

Next, we examined morphological changes in KG1a and KG1 cells following treatment with DMSO or ATRA. Cells were either untreated or treated with DMSO or ATRA for five days and then stained with Wright-Giemsa solutions. No granulocytic morphology was seen in KG1a cells after treatment with either ATRA or DMSO (**Figure 4B**). In contrast, ATRA treatment induced morphological changes in KG1 cells, including the appearance of band cells containing azurophil granules (**Figures 5B, C**). The percentage of azurophil granule-positive KG1 cells increased from 2.9 ± 0.4% to 54.6 ± 2.8% of the total cell population (mean ± SE, 6 independent experiments). In contrast, neither ATRA-treated KG1a cells nor DMSO-treated KG1 cells showed an increase in azurophil granule-positive cells.

#### 3.3.3 DIM21 induced apoptosis in DMSO-treated KG1 but not KG1a cells

DIM21 induced early apoptosis in both DMSO- and ATRA-treated HL-60 cells (**Figure 1B**). We investigated whether DIM21 induces early apoptosis in DMSO- or ATRA-treated KG1a and KG1 cells. In KG1a cells, DIM21 did not induce early apoptosis under any treatment condition, including DMSO, ATRA, or ATRA plus DIM21 (**Figures 4C, D**, **Supplementary Figure S4A**). In contrast, DIM21 induced early apoptosis in KG1 cells regardless of treatment (**Figures 5C–E**, **Supplementary Figure S4B**).

#### 3.3.4 DIM21 enhanced late apoptosis in ATRA-treated KG1 cells

**Figure 6:**
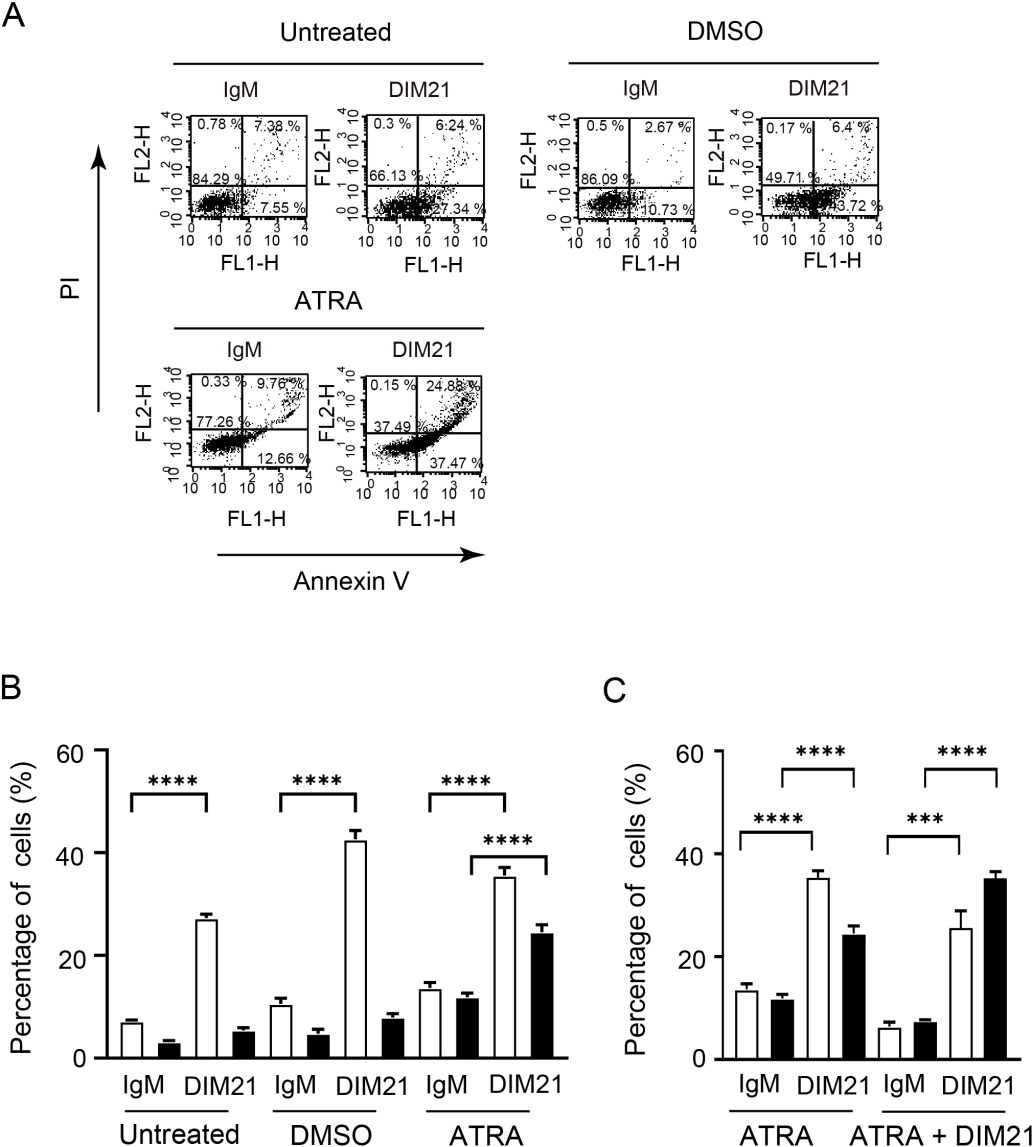
DIM21-induced apoptosis in KG1 cells treated with DMSO or ATRA. **(A)** Apoptosis induced in DMSO- or ATRA-treated KG1 cells. KG1 cells were treated without (Untreated) or with DMSO (1.3%) or ATRA (1 μM) for six days. Cells were then incubated with 4 μg/ml IgM (control) or DIM21 for 4 h. Apoptotic cells (Annexin V^+^+PI^-^, lower right quadrant) and late apoptotic/dead cells (Annexin V^+^+PI^+^, upper right quadrant) were analyzed by flow cytometry. Percentages of cells in each quadrant are shown. Representative images from 9–30 independent experiments are shown. **(B)** Increased cell death in ATRA-treated KG1 cells following DIM21 treatment. KG1 cells were treated as described and incubated with 4 μg/ml DIM21 or IgM for 4 h. Flow cytometry was used to quantify early-apoptotic cells (Annexin V^+^+PI^-^) and late-apoptotic/dead cells (Annexin V^+^+PI^+^). Apoptosis and cell death induced by DIM21 were compared among untreated, DMSO-, and ATRA-treated KG1 cells. Data are expressed as percentages of Annexin V^+^+PI^-^ cells (white bars) and Annexin V^+^+PI^+^ cells (black bars). Results represent the mean ± SE of 9–30 independent experiments. ****P < 0.0001. **(C)** ATRA plus DIM21 enhances cell death compared to ATRA alone. KG1 cells were treated with ATRA (1 μM) or ATRA + DIM21 (2 μg/ml) for six days. DIM21-induced apoptosis and cell death were assessed by flow cytometry, as shown in **(A)**. Early apoptotic cells (Annexin V^+^+PI^-^, lower right quadrant) and late apoptotic/dead cells (Annexin V^+^+PI^+^, upper right quadrant) were quantified. Ratios of Annexin V^+^+PI^-^cells (white bars) and Annexin V^+^+PI^+^ cells (black bars) were compared between ATRA-treated and ATRA + DIM21-treated groups. Data represent the mean ± SE of 14 independent experiments. ***P < 0.001, ****P < 0.0001.

To further characterize the effect of DIM21 on KG1 apoptosis, we examined its influence on early and late apoptosis with and without differentiation inducers (**Figure 6**). In untreated or DMSO-treated KG1 cells, incubation with DIM21 for 4 h induced early but not late apoptosis (**Figures 6A, B**). In contrast, DIM21 treatment enhanced both early and late apoptosis in ATRA-treated KG1 cells (**Figure 6**, **Supplementary Figure S4B**). In particular, DIM21 markedly enhanced late apoptosis in ATRA plus DIM21 treated KG1 cells (**Figure 6C**, **Supplementary Figure S4B**). These results indicate that high PtdGlc expression in KG1 cells is important in their apoptosis.

### 3.4 Characterization of DIM21-induced apoptosis in KG1 cells

#### 3.4.1 DIM21-induced apoptosis in KG1 cells involves caspase activation

**Figure 7:**
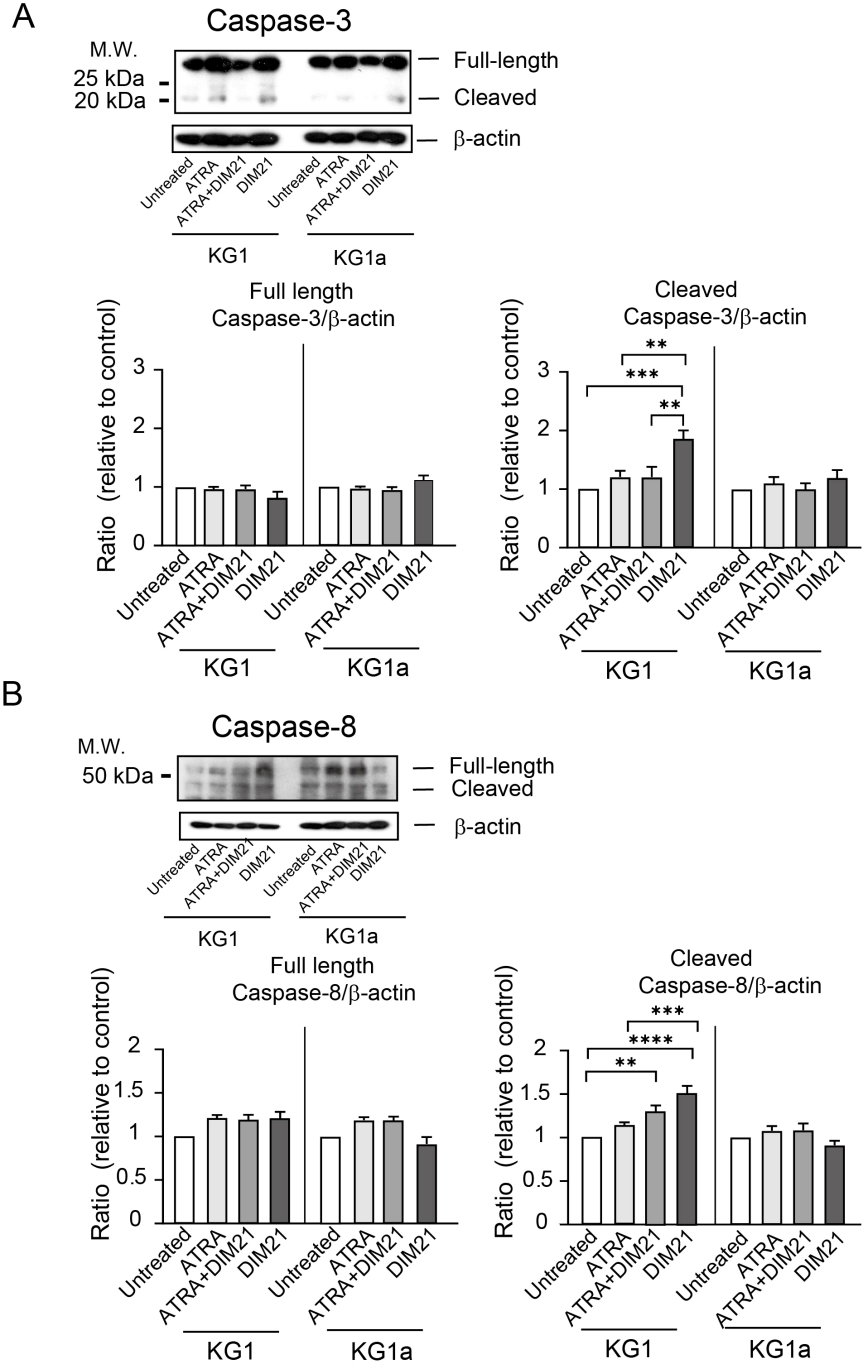
Activation of Caspase-3 and -8 in ATRA plus DIM21- or DIM21-treated KG1 cells but not in KG1a cells. **(A)** Caspase-3 activation. KG1 or KG1a cells were treated as described in the text. After treatment, cells were harvested, washed, and lysed with RIPA buffer. Proteins (20 μg) were subjected to SDS-PAGE, transferred to an Immobilon membrane, and analyzed by Western blotting with an anti-caspase-3 antibody. Expression levels were normalized to those of untreated cells. Representative images and quantifications from 6–8 independent experiments are shown. Data are presented as the mean ± SE. **P < 0.01, ***P < 0.0005. **(B)** Caspase-8 activation. Proteins from treated KG1 and KG1a cells were analyzed as described above using an anti-caspase-8 antibody. Expression ratios were calculated relative to untreated cells. Representative images and data from 6–8 independent experiments are presented as the mean ± SE. **P < 0.005, ***P < 0.0005, ****P < 0.0001.

DIM21 induces apoptosis in peripheral human neutrophils without Fas ligands (FasL) via caspase-3, -8, and -9 (19). To determine whether caspases are involved in DIM21-induced apoptosis of KG1 cells, we treated cells with caspase-specific inhibitors for 16 h and analyzed the effects on apoptosis. Inhibitors of caspase-3, -8, and -9 significantly attenuated apoptosis (**Supplementary Figure S5**), indicating that these caspases contribute to DIM21-induced apoptosis. To further confirm caspase involvement, we analyzed cell lysates from KG1 and KG1a cells under different treatment conditions (**Figure 7**). In KG1 cells, caspase-3 activation was detected in DIM21-treated cells, while caspase-8 activation was observed in both DIM21- and ATRA plus DIM21-treated cells. However, no significant caspase-9 activation was detected (data not shown). These findings suggest PtdGlc-mediated apoptosis in KG1 cells may not be fully dependent on the Fas/caspase-8 signaling cascade. As expected, KG1a cells showed no activation of caspase-3 or -8 under any treatment condition. While western blot analysis showed caspase-8 cleavage in all KG1a samples, the cleaved form remained unchanged (**Figure 7B**). Given that KG1a cells express Fas, Fas-independent caspase-8 self-cleavage may occur in these cells.

#### 3.4.2 DIM21-induced apoptosis in KG1 cells is independent of ROS and the PI3K/AKT signaling pathway

**Figure 8:**
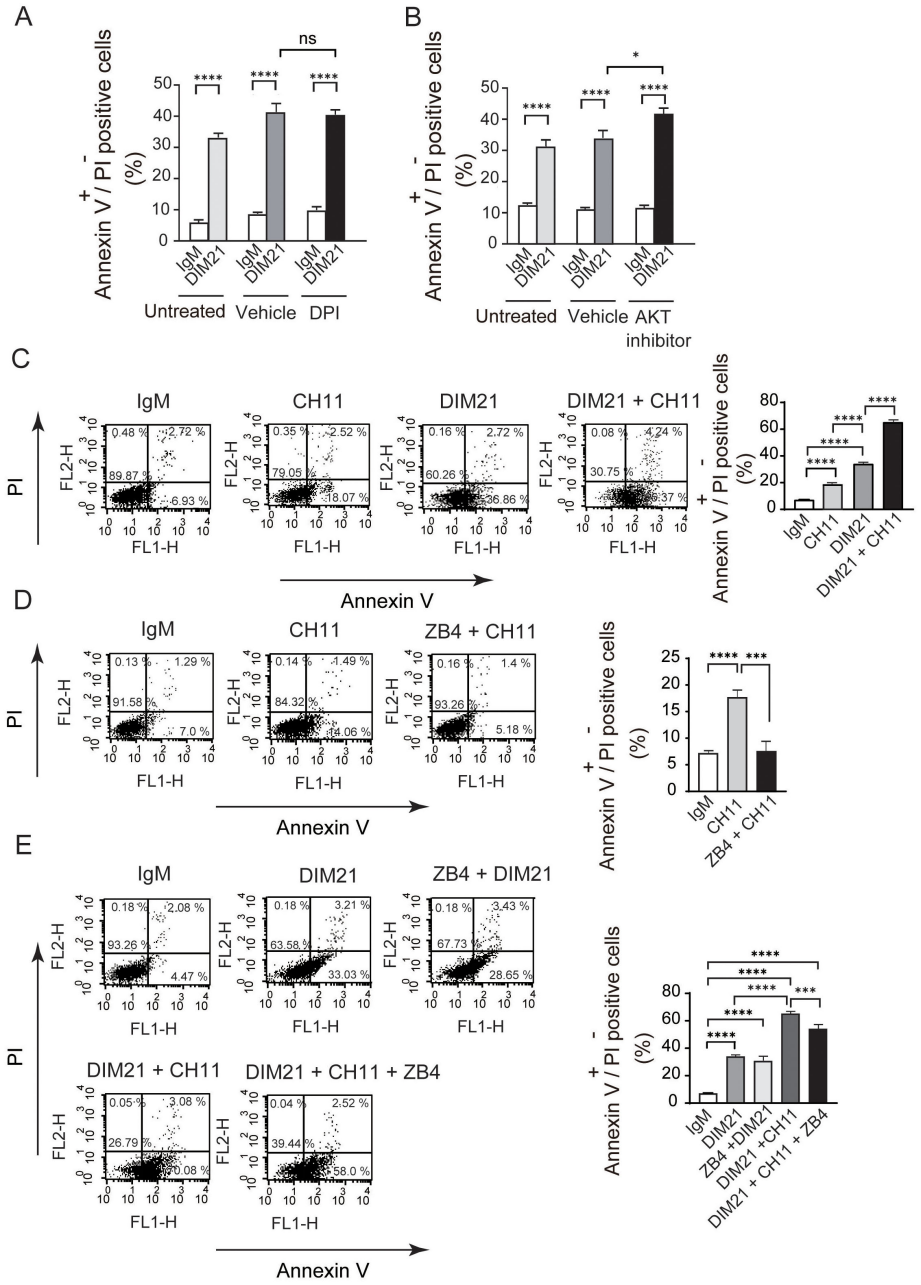
Apoptosis induced by anti-PtdGlc or anti-Fas antibodies in KG1 Cells. **(A)** NADPH oxidase-mediated ROS does not contribute to DIM21-induced apoptosis. KG1 cells were pretreated with 10 μM DPI or vehicle (DMSO, 2.83 μl) for 1 h, followed by 4 μg/ml DIM21 or IgM for 4 h. Apoptotic cells (Annexin V^+^+ PI^-^, lower right quadrant) were analyzed using an Annexin V- binding assay. Data represent the mean ± SE of 5 independent experiments. ****P < 0.0001. **(B)** AKT inhibitor enhances DIM21-induced apoptosis. KG1 cells were treated with 5 μM AKT1/2 inhibitor or vehicle (DMSO, 1 μl) in the presence of 4 μg/ml DIM21 or IgM for 4 h. Apoptotic cells were analyzed as described above. Data represent the mean ± SE of 6–8 independent experiments. *P < 0.05, ****P < 0.0001. **(C)** Synergistic effect of DIM21 on anti-Fas-induced apoptosis. KG1 cells were incubated with IgM (4 μg/ml), anti-Fas antibody (CH-11, 5 μg/ml), DIM21 (4 μg/ml), or DIM21 + anti-Fas antibody for 4 h. Annexin V^+^+PI^-^ cells were analyzed by flow cytometry. Data represent the mean ± SE of 14 independent experiments. ****P < 0.0001. **(D)** Anti-Fas-induced apoptosis is blocked by ZB4 (Fas antagonist). KG1 cells were incubated with or without ZB4 (10 μg/ml) for 2 h at 37°C, followed by anti-Fas antibody (CH-11, 5 μg/ml) or IgM (5 μg/ml) for 4 h. Annexin V^+^+PI^-^ cells were analyzed. Data represent the mean ± SE of 8 independent experiments. ***P < 0.0005, ****P < 0.0001. **(E)** DIM21-induced apoptosis is not blocked by ZB4. KG1 cells were incubated with or without ZB4 (10 μg/ml) for 2 h at 37°C, followed by treatment with DIM21 (4 μg/ml), IgM (4 μg/ml), or anti-Fas antibody (CH-11, 5 μg/ml) for 4 h. Apoptotic cells were analyzed as described above. Data represent the mean ± SE of 7 independent experiments. ***P < 0.001, ****P < 0.0001.

Recent studies suggest reactive oxygen species (ROS) contribute to apoptosis, including caspase activation, in myeloid cells (38–40). To assess whether ROS are involved in DIM21-induced apoptosis, we pretreated KG1 cells with DPI, a potent nicotinamide adenine dinucleotide phosphate (NADPH) oxidase inhibitor that also suppresses ROS production. DPI had no affect on DIM21-induced apoptosis (**Figure 8A**), consistent with findings in peripheral neutrophils (19). These results suggest ROS are not involved in DIM21-induced apoptosis in KG1 cells.

The phosphatidylinositol-3 kinase (PI3K)/AKT signaling pathway is often activated in AML, and PI3K/AKT inhibitors have demonstrated potent anti-leukemic activity (41). To examine the effect of AKT inhibitors on DIM21-induced apoptosis, we treated KG1 cells with an AKT inhibitor in combination with DIM21 or control IgM, using DMSO as a vehicle control. The AKT inhibitor significantly enhanced DIM21-induced apoptosis (**Figure 8B**). This is consistent with previous reports showing that the PI3K inhibitor LY294002 does not inhibit DIM21-induced apoptosis in neutrophils (19). These results suggest the PI3K/AKT pathway is not involved in PtdGlc-mediated apoptosis in KG1 cells. In fact, sequential treatment with AKT1/2 inhibitors further enhanced apoptosis in ATRA-treated KG1 cells (data not shown).

#### 3.4.3 DIM21-induced apoptosis of KG1 cells is independent of Fas

To characterize the role of Fas in DIM21-induced apoptosis, we examined apoptosis induced by the anti-Fas monoclonal antibody CH11. CH11 mimics FasL, leading to the formation of the death-inducing signaling complex (42). As shown in **Figure 8C**, CH11-induced apoptosis in KG1 cells at levels approx. 50% lower than those induced by DIM21. DIM21 plus anti-Fas antibody treatment synergistically enhanced apoptosis. However, pretreatment with neutralizing anti-Fas antibody ZB4 blocked CH11-induced apoptosis but not DIM21-induced apoptosis, suggesting that DIM21-mediated apoptosis occurs independently of the Fas/FasL signaling pathway (**Figures 8D, E**). Fas expression does not always correlate with apoptosis in AML cell lines (43, 44). Indeed, KG1a cells did not undergo apoptosis following CH11 treatment (**Supplementary Figure S6**), despite expressing Fas on their surfaces (**Figure 4A**).

## 4 Discussion

A proper balance between cell death and survival is essential for maintaining hematopoietic homeostasis. In this study, we demonstrated that high expression of phosphatidylglucoside (PtdGlc) plays a critical role in apoptosis induction in human AML cells. Among leukocytes, neutrophil apoptosis is a well-established mechanism that contributes to the resolution of inflammation and the regulation of immune homeostasis. Human peripheral neutrophils highly express PtdGlc on their plasma membrane, and previous studies have implicated PtdGlc in both neutrophil apoptosis and ATRA-like differentiation of HL-60 cells (6, 7, 19). However, the molecular mechanism linking differentiation and apoptosis in myeloid cells remains incompletely understood.

Our data showed that undifferentiated HL-60 cells, which exhibited relatively low PtdGlc expression, did not undergo apoptosis in response to DIM21. In contrast, HL-60 cells differentiated with either DMSO or ATRA showed increased PtdGlc expression and became susceptible to DIM21-induced apoptosis. These findings suggest that elevated PtdGlc levels contribute to apoptosis sensitivity, although DIM21 itself did not induce differentiation. Consistent with earlier reports (45–47), ATRA treatment alone led to G0/G1 arrest in HL-60 cells and reduced S-phase cell populations without triggering apoptosis. However, when ATRA treatment was combined with DIM21, we observed enhanced G0/G1 arrest, a decrease in G2/M and S-phase populations, and significant induction of apoptosis. These observations imply that PtdGlc-mediated signaling may influence both cell cycle arrest and apoptotic pathways in differentiating myeloid cells.

In KG1 cells, PtdGlc was highly expressed regardless of differentiation stimuli, and these cells were susceptible to DIM21-induced apoptosis. Conversely, KG1a cells lacked surface PtdGlc expression, which remained low following DMSO or ATRA treatment, and showed resistance to DIM21-induced apoptosis. These results highlight the essential role of PtdGlc expression in enabling apoptosis, regardless of the differentiation state. The differential responsiveness between KG1 and KG1a cells may be due to intrinsic differences in PtdGlc biosynthesis, membrane trafficking, lipid composition, or glycosylation profiles. All of which could potentially affect the formation or function of PtdGlc-associated membrane microdomains.

Mechanistically, we demonstrated that DIM21-induced apoptosis in KG1 cells is caspase-dependent, as it was inhibited by caspase-3, -8, and -9 inhibitors, but unaffected by either the NADPH oxidase inhibitor DPI or a neutralizing Fas antibody. These findings indicate that PtdGlc-mediated apoptosis operates independently of both ROS and Fas signaling. This is consistent with previous reports showing that retinoids can upregulate tumor necrosis factor-related apoptosis-inducing ligand (TRAIL), which is an apoptosis-inducing ligand capable of functioning independently of ROS and Fas pathways (48). Although TRAIL was not directly assessed in this study, its reported involvement in retinoid-induced apoptosis suggests a possible contribution to the DIM21-induced pathway, particularly under ATRA-treated conditions. Future studies are needed to evaluate the potential role of TRAIL or other death ligands in this context.

Our previous work also indicated that PtdGlc signaling is independent of the PI3K/AKT pathway (19), further supporting the notion that PtdGlc engages alternative, non-canonical apoptotic regulators. Elucidating these downstream effectors will be critical for defining the molecular identity of this pathway. In particular, detailed analysis of PtdGlc-enriched lipid microdomains in DIM21-treated AML cells will be essential to understand how these domains orchestrate apoptosis signaling.

In line with prior findings, Fas-mediated apoptosis does not always correlate with Fas receptor expression levels, and functional Fas ligand expression also contributes to apoptotic sensitivity (43, 44). In our study, anti-Fas antibody weakly induced apoptosis in KG1 cells, whereas co-treatment with DIM21 synergistically enhanced cell death. Notably, pretreatment with a neutralizing Fas antibody blocked anti-Fas-induced apoptosis but did not affect DIM21-mediated apoptosis, suggesting that Fas and PtdGlc may function in parallel or partially overlapping pathways, potentially localized within shared membrane regions.

Clinically, ATRA is well established as a treatment for acute promyelocytic leukemia (APL), particularly when used in combination with arsenic trioxide (ATO), a ROS-generating pro-apoptotic agent (49). However, ATRA monotherapy does not induce apoptosis in all cases (30, 50), and ATRA/ATO combination therapy can lead to adverse effects such as hepatotoxicity, leukocytosis, differentiation syndrome, and cytokine release (51, 52). Notably, ATO is also associated with cardiac and renal toxicities (53–55). Given that DIM21-induced apoptosis occurs via a ROS-independent pathway, targeting PtdGlc could represent an alternative therapeutic strategy that bypasses these side effects, although *in vivo* studies will be necessary to evaluate its translational relevance.

In conclusion, our findings demonstrate that PtdGlc plays a key role in regulating apoptosis in neutrophilic AML cell lines, acting through a caspase-dependent, ROS-independent mechanism. DIM21-mediated activation of PtdGlc-enriched membrane domains may provide a novel strategy to enhance differentiation-associated apoptosis, especially in ATRA- or ATO-resistant contexts. While our *in vitro* data offer compelling insights, further investigation using patient-derived samples and *in vivo* models will be essential to validate the therapeutic potential of PtdGlc. Moreover, assessing the prognostic significance of PtdGlc expression and its relationship to treatment responsiveness may inform its future application as a biomarker and therapeutic target in AML.

## Data Availability

The original contributions presented in the study are included in the article/**Supplementary Material**. Further inquiries can be directed to the corresponding authors.
