# Supplementary figures and images for: Phosphatidylglucoside regulates apoptosis of human neutrophilic lineage cells
Source: Front Immunol. 2025 May 27;16:1597423. doi: 10.3389/fimmu.2025.1597423 (PMC12149147; doi:10.3389/fimmu.2025.1597423)

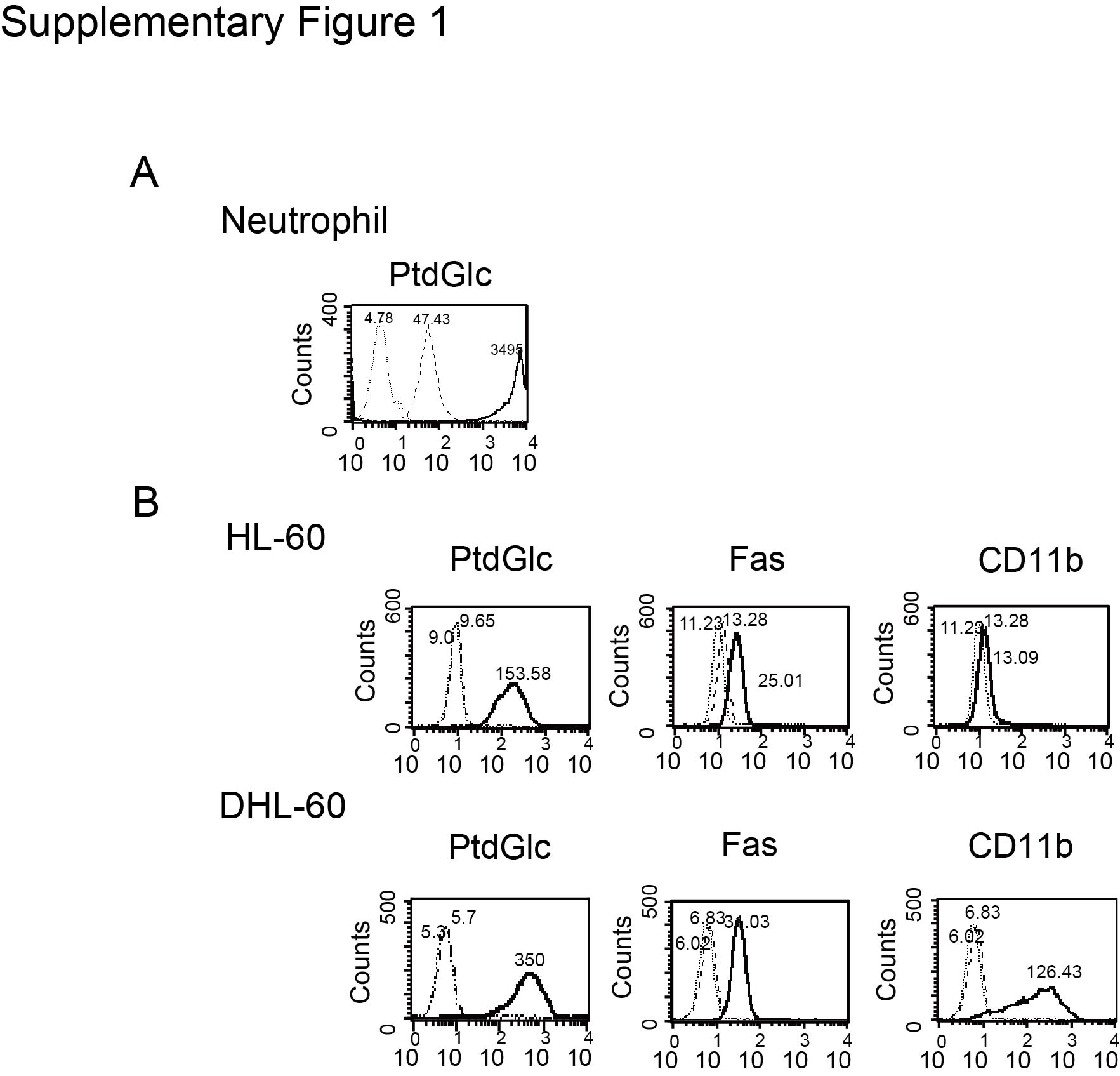

Supplement: Supplementary file 1 [file Image1.jpeg]

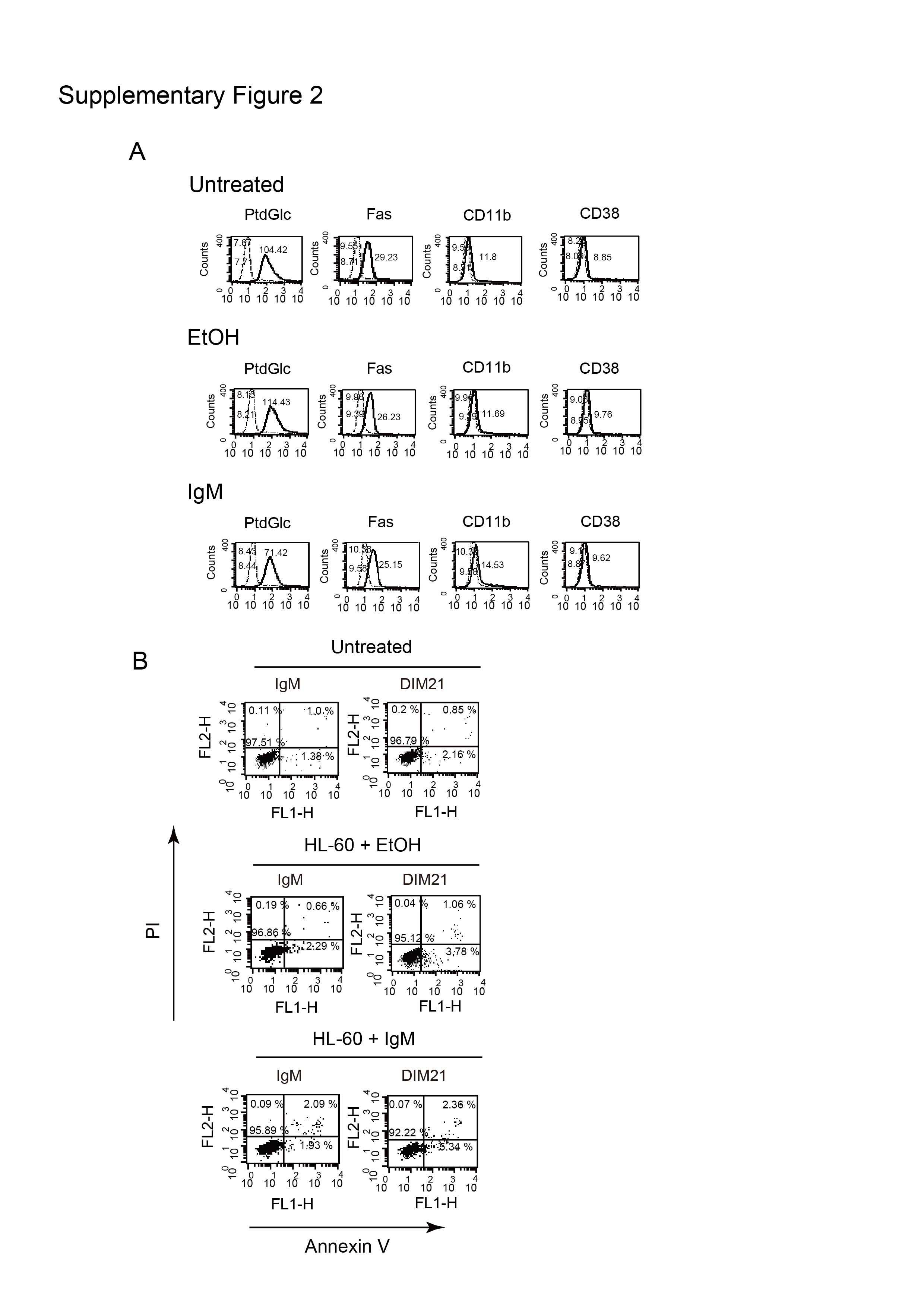

Supplement: Supplementary file 2 [file Image2.jpeg]

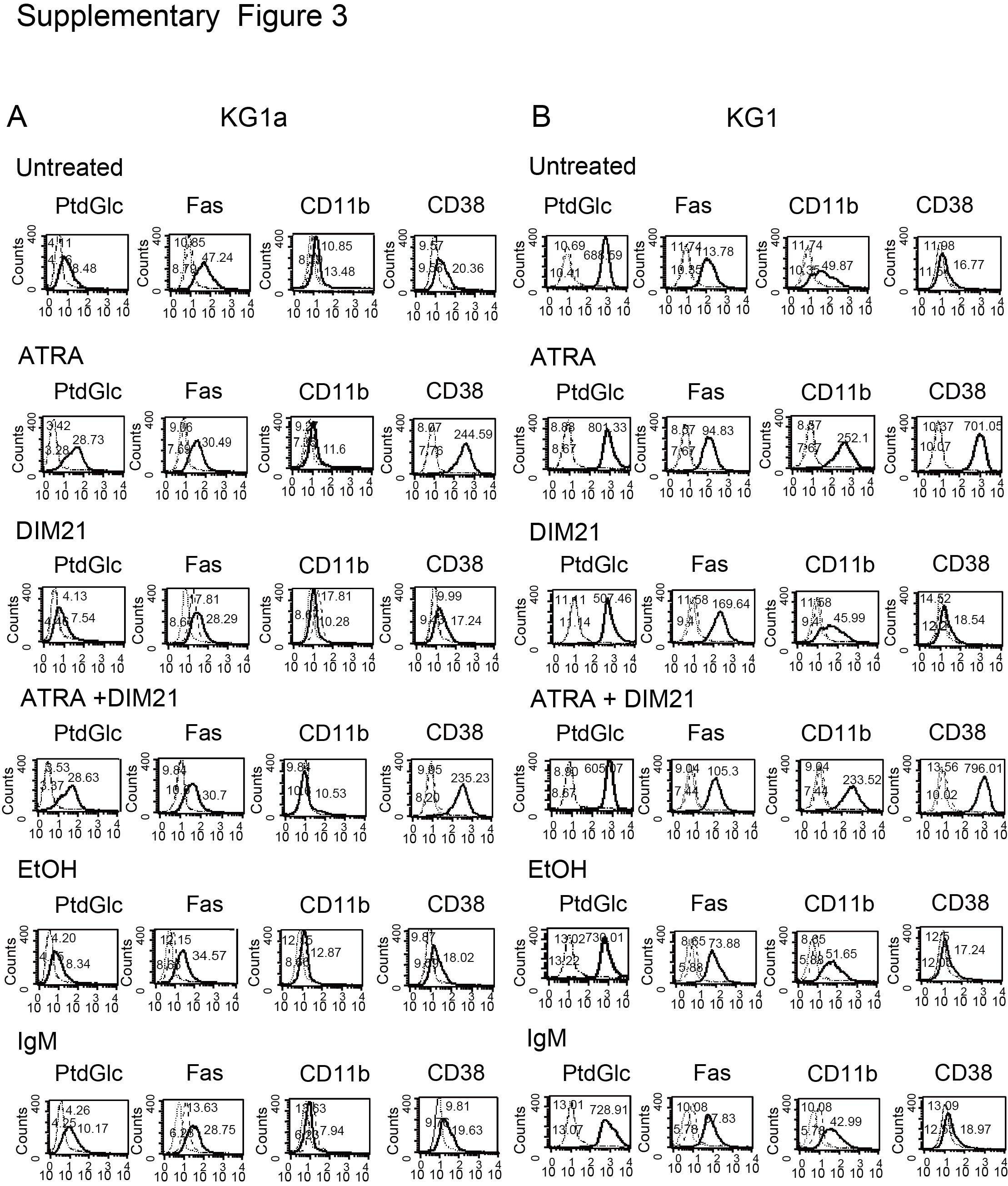

Supplement: Supplementary file 3 [file Image3.jpeg]

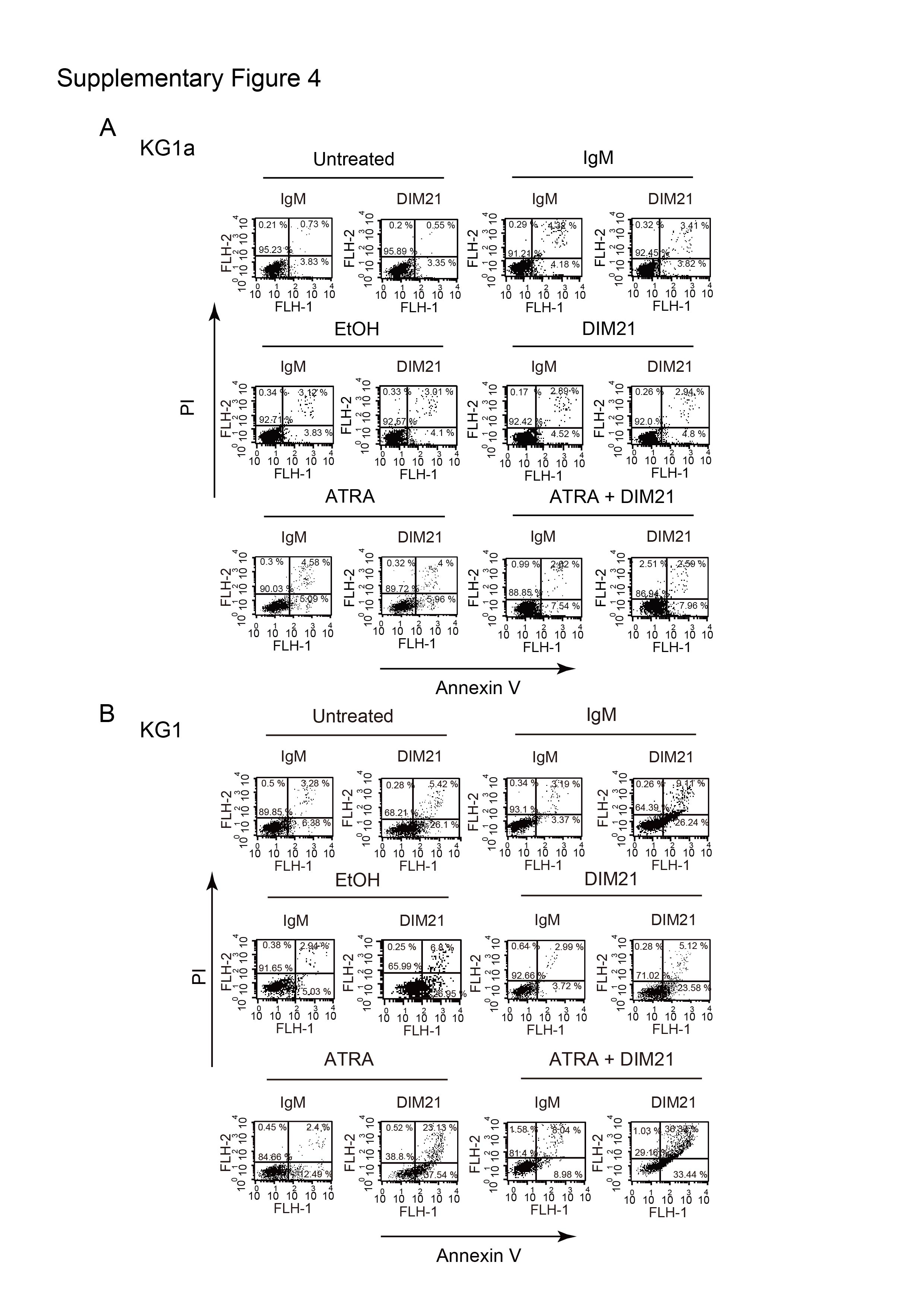

Supplement: Supplementary file 4 [file Image4.jpeg]

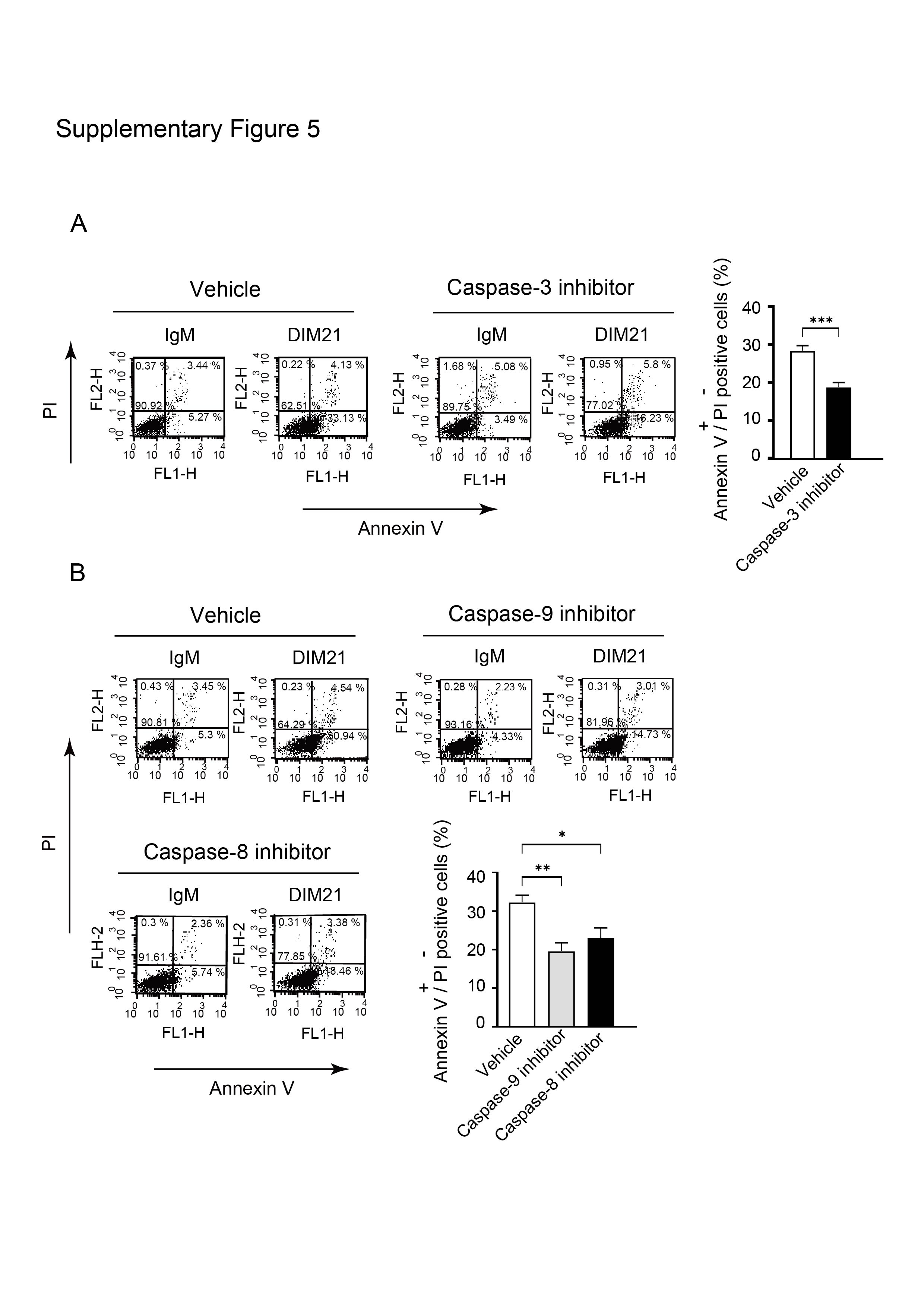

Supplement: Supplementary file 5 [file Image5.jpeg]

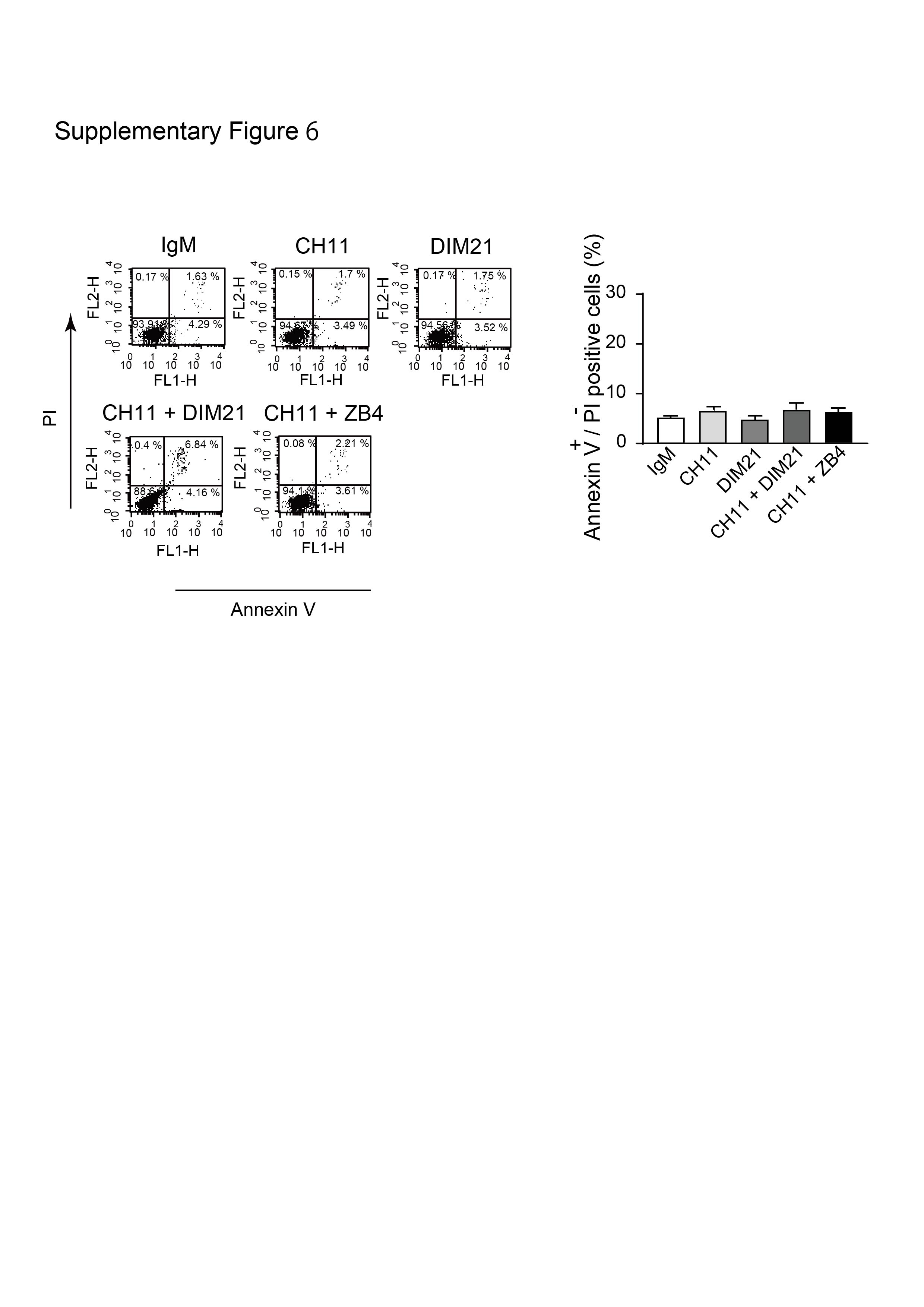

Supplement: Supplementary file 6 [file Image6.jpeg]
