# Supplementary material for: Phosphatidylglucoside regulates apoptosis of human neutrophilic lineage cells
Source: Front Immunol. 2025 May 27;16:1597423. doi: 10.3389/fimmu.2025.1597423 (PMC12149147; doi:10.3389/fimmu.2025.1597423)
Supplement: Supplementary file 7 [file DataSheet1.docx]

**Title**

**Phosphatidylglucoside regulates apoptosis of human neutrophilic lineage cells**

**List of authors**:

Noriko Yokoyama^1^*^,^ Roudy Chiminch Ekyalongo^1^, Madoka Kage^1,2^, Kei Hanafusa^1, 3^, Hitoshi Nakayama^1, 4, 5^, Yoshio Hirabayashi^1,6^ Kenji Takamori^1^, and Kazuhisa Iwabuchi^1, 3^*

^1^ Institute for Environmental and Gender Specific Medicine, Juntendo University Graduate School of Medicine, Urayasu Chiba 279-0021, Japan.

^2^ Laboratory of Dermatological Physiology, Faculty of Pharmacy and Pharmaceutical Sciences, Josai University, 1-1 Keyakidai, Sakado, Saitama 350-0295, Japan

^3^ Laboratory of Biochemistry, Faculty of Pharmacy, Juntendo University, Urayasu Chiba 279-0013, Japan

^4^ Infection Control Nursing, Juntendo University Graduate School of Health Care and Nursing, Urayasu, Chiba 279-0023, Japan.

^5^ Laboratory of Biochemistry, Juntendo University Faculty of Health Care and Nursing, Urayasu, Chiba 279-0023, Japan.

^6^ Cellular Informatics Lab, RIKEN, 2-1 Hirosawa Wako city, Saitama 351-0198 Japan

*: Corresponding Authors:

Noriko Yokoyama, [n-yokoyama@juntendo.ac.jp](mailto:n-yokoyama@juntendo.ac.jp),

Kazuhisa Iwabuchi, [iwabuchi@juntendo.ac.jp](mailto:iwabuchi@juntendo.ac.jp),

Table of contents:

1. Supplementary Table S1
2. Supplementary methods
3. Supplementary Figure S1
4. Supplementary Figure S2
5. Supplementary Figure S3
6. Supplementary Figure S4
7. Supplementary Figure S5
8. Supplementary Figure S6

Supplementary Materials:

**Supplementary Table S1. List of antibodies used in the study**

| Antibodies | Company | Location |
| --- | --- | --- |
| An anti-beta actin antibody | Sigma-Aldrich | St. Louis, MO, USA |
| Alexa 488-conjugated Annexin V | Invitrogen | Thermo Fisher Scientific, Waltham, MA, USA |
| Anti-caspase-3 antibody | Cell Signaling Technology | Danvers, MA, USA |
| Anti-caspase-8 antibody | Cell Signaling Technology | Danvers, MA, USA |
| Anti-caspase-9 antibody | Cell Signaling Technology | Danvers, MA, USA |
| Anti-human Fas antibody (CD95, clone CH-11) | MBL | Tokyo, Japan |
| Anti-human Fas antibody (CD95, clone ZB4) | MBL | Tokyo, Japan |
| PE-labeled anti-mouse IgG1 κ isotype | eBioscience, Inc | San Diego, CA, USA |
| PE-labeled anti-human CD11b | eBioscience, Inc | San Diego, CA, USA |
| PE-labeled anti-human Fas | eBioscience, Inc | San Diego, CA, USA |
| PE-labeled anti-human CD14 | eBioscience, Inc | San Diego, CA, USA |
| CD95 antibody (Dx2) | eBioscience, Inc | San Diego, CA, USA |
| Alexa 488-labeled anti-human CD38 | BioLegend | San Diego, CA, USA |
| Alexa 488-labeled anti-mouse IgG1 κ isotype | BioLegend | San Diego, CA, USA |

**Supplementary methods:**

**Preparation of neutrophils.**

Ethical approval for obtaining blood from healthy human volunteers was provided by the Ethics Review Board of Juntendo University Faculty of Medicine (Authorization number: 2020235). All research was performed in accordance with the Declaration of Helsinki and relevant guidelines/regulations. Peripheral blood was obtained from healthy volunteers who provided written informed consent. Peripheral blood was used to isolate human neutrophils using Polymorphprep^TM^ (Nycomed Pharma; Oslo, Norway), according to the manufacturer’s instructions as described before (1).

**Supplementary Figure Legends:**

**Supplementary Figure S1. Expressions of PtdGlc, Fas, and CD11b upon differentiation.** **(A) High expression of PtdGlc in neutrophils.** Human neutrophils were isolated from the peripheral blood of a healthy volunteer. Cells (2.5 x 10^6^ cells/ml) were stained with Alexa488-conjugated DIM21 or Alexa488-conjugated mouse IgM. Washed cells were analyzed by flow cytometry as described in *Materials and Methods*. Histograms show DIM21 (thick lines), normal IgM or (broad dashed lines, ----) and untreated cells by narrow dashed lines (^……^). Numbers indicate geometric mean fluorescence intensity. **(B) Upregulation of PtdGlc, Fas, and CD11b expressions in DMSO-induced differentiation of HL-60 cells (DHL-60).** HL-60 cells were cultured without (HL-60) or with 1.3 v/v% DMSO (DHL-60) for five days. Cells (2.5 x 10^6^ cells/ml) were stained with Alexa488-conjugated DIM21, Alexa488-conjugated mouse IgM, PE-anti-human Fas IgM, Alexa488- or PE-conjugated mouse IgM, PE-anti-CD11b IgG, or PE-normal IgG1 κ. Washed cells were analyzed by flow cytometry as described in *Materials and Methods*. The thick lines represent PtdGlc, Fas, and CD11b, respectively. Negative controls were shown as broad dashed lines (-----), and unstained cells were shown as thin dashed lines (^……^). The numbers represent the geometric mean of fluorescence intensity. Representative results are shown from 3 independent experiments.

**Supplementary Figure S2. Multiple molecule expression and DIM21-induced apoptosis in HL-60 cells treated with EtOH or IgM.** HL-60 cells were untreated or treated with either 2 μg/ml normal IgM or 0.02% EtOH as a vehicle for six days and expressions of multiple molecules and DIM21-induced apoptosis were examined. **(A) Expressions of multiple molecules in HL-60 cells treated with either EtOH or IgM.** Cells (2.5 x 10^6^ cells/ml) were incubated with either Alexa488-conjugated DIM21, Alexa488-conjugated normal mouse IgM, PE-anti-human Fas, PE-normal mouse IgG1 κ, PE-anti-human CD11b, Alexa 488-conjugated normal mouse IgG, or Alexa 488-conjugated anti-human CD38 mouse IgG. After staining, cells were washed, and analyzed by flow cytometry. The thick lines represented PtdGlc, Fas, CD11b, and CD38, respectively. Negative controls were shown as broad dashed lines (-----) and unstained cells were shown as thin dashed lines (^……^). The numbers represent the geometric mean of fluorescence intensity. Representative results from 6 independent experiments are shown. **(B) DIM21-induced apoptosis in HL-60 cells treated with either EtOH or IgM.** HL-60 cells were either untreated or treated as indicated for six days as shown in (A). Cells were treated with 4 μg/ml IgM or DIM21 for 4h. Then, the apoptosis assay was performed by flow cytometry as described in *Materials and Methods*. Early apoptotic cells (Annexin V^+^+PI^-^) are shown in the lower right quadrant, while late apoptotic/dead cells (Annexin V^+^+PI^+^) appear in the upper right quadrant. Quadrant numbers (%) indicate the percentage of cells in each quadrant. Results shown represent 3-6 independent experiments.

**Supplementary Figure S3.** **ATRA** **induces KG1 cells to differentiate but not KG1a cells. (A) Induction of CD38 expression in ATRA-treated KG1a cells*.*** KG1a cells were treated without (Untreated) or with either ATRA (1 μM), 2 μg/ml DIM21, ATRA + DIM21, 0.02% EtOH as a solvent control, or 2 μg/ml normal IgM for six days. Expressions of PtdGlc, Fas, CD11b, and CD38 were analyzed as described in *Materials and Methods.* The thick lines represent PtdGlc, Fas, CD11b, and CD38, respectively. Negative control antibodies were shown as broad dashed lines (-----) and unstained cells were shown as thin dashed lines (^……^). Numbers indicate geometric means of fluorescence intensity. Representative results from 14 independent experiments are shown. **(B) Upregulation of PtdGlc, CD11b, and CD38 expression in ATRA-treated KG1 cells.** KG1 cells were treated without or with either 2 μg/ml DIM21, 1 μM ATRA, ATRA + DIM21, 0.02% EtOH, or 2 μg/ml normal IgM for six days. Expressions of PtdGlc, Fas, CD11b, and CD38 were determined by flow cytometry. Histograms show PtdGlc, Fas, CD11b, and CD38 expression (thick lines), with negative control antibodies shown as broad dashed lines (-----) and untreated cells shown as thin dashed lines (^……^). Numbers represent the geometric mean of fluorescence intensity. Representative results from 5-8 independent experiments are shown.

**Supplementary Figure S4. DIM21-induced apoptosis in ATRA-treated KG1a or KG1 cells.** **(A) DIM21 fails to induce apoptosis in ATRA-treated KG1a cells.** KG1a cells were treated without (Untreated) or with either ATRA (1 μM), 2 μg/ml DIM21, ATRA + DIM21, vehicle (EtOH), or normal IgM for six days, followed by incubated with 4μg/ml normal IgM or DIM21 for 4 h at 37^o^C. Then, Annexin V-binding assay was performed as described in *Materials and Methods.* Early apoptotic cells (Annexin V^+^+PI^-^) appear in the lower right quadrant, while late apoptotic/dead cells (Annexin V^+^+PI^+^) appear in the upper right quadrant. The results shown are representative of 6-14 independent results. **(B) Increased cell death in ATRA-treated KG1 cells after DIM21 treatment.** KG1 cells were treated without or with either ATRA (1 μM), ATRA plus DIM21, 2 μg/ml DIM21, EtOH, or IgM for six days. Cells were treated with either 4μg/ml with normal IgM or DIM21 for 4 h at 37^o^C. Annexin V-binding assay was performed as described in *Materials and Methods.* Early apoptotic cells (Annexin V^+^+PI^-^) appear in the lower right quadrant, while late apoptotic/dead cells (Annexin V^+^+PI^+^) appear in the upper right quadrant. The results shown are representative of 14 independent results.

**Supplementary Figure S5.** **Caspase-3, -8, and -9 are activated during DIM21-induced apoptosis in KG1 cells.** KG1 cells (2.5 x 10 ^6^ cells/ml) were pretreated with either 10 μM caspase-3 inhibitor (A), 10 μM caspase-8 inhibitor, or caspase-9 inhibitor (B) for 16 h. Treated cells were further incubated at 4 μg/ml normal mouse IgM or DIM21 for 4h. Then, Annexin V-binding assay was carried out using flow cytometry, as described in *Materials and Methods*. Early apoptotic cells (Annexin V^+^+PI^-^) are shown in the lower right quadrant. The percentages of early apoptotic of vehicles (white bars) or inhibitor-treated cells (gray or black bars) were quantified after treatment with DIM21 or IgM. Representative results from 4-8 independent experiments are shown. P*< 0.05, P** < 0.005, P***<0.0005.

**Supplementary Figure S6. Anti-Fas antibody failed to induce apoptosis in KG1a cells.** KG1a cells (2.5 x 10 ^6^ cells/ml) were incubated with either 4 μg/ml nomal IgM, 5 μg/ml anti-Fas IgM CH-11, 4 μg/ml DIM2, or DIM21 + anti-Fas antibody for 37 ^o^C 4h. In some cases, KG1a cells were preicubated with 10 μg/ml anti-Fas neutralizing IgM ZB4 at 37 ^o^C for 2h, followed by incubation with 5 μg/ml CH11 for 4h at 37 ^o^C. Then, the Annexin V-binding to the cells was analyzed by flow cytometry as described in *Materials and Methods*. The percentages of early apoptotic cells (Annexin V^+^+PI^-^) of the IgM, CH11, DIM21, CH11 + DIM21, CH11 + ZB4 cells were quantified. Numbers represent geometric mean fluorescence intensity. Data represent the mean±SE of 5-16 independent experiments.

**Supplementary Figure References**

1. Nakayama, H., Kurihara, H., Morita, Y. S., Kinoshita, T., Mauri, L., Prinetti, A. *et al.* (2016) Lipoarabinomannan binding to lactosylceramide in lipid rafts is essential for the phagocytosis of mycobacteria by human neutrophils Sci Signal **9**, ra101 10.1126/scisignal.aaf1585
